# Uniform Sb_2_S_3_ optical coatings by chemical spray method

**DOI:** 10.3762/bjnano.10.18

**Published:** 2019-01-15

**Authors:** Jako S Eensalu, Atanas Katerski, Erki Kärber, Ilona Oja Acik, Arvo Mere, Malle Krunks

**Affiliations:** 1 Laboratory of Thin Film Chemical Technologies, Department of Materials and Environmental Technology, Tallinn University of Technology, Ehitajate tee 5, Tallinn 19086, Estonia

**Keywords:** antimony sulfide, thin films, ultrasonic spray, vacuum annealing, Volmer–Weber growth

## Abstract

Antimony sulfide (Sb_2_S_3_), an environmentally benign material, has been prepared by various deposition methods for use as a solar absorber due to its direct band gap of ≈1.7 eV and high absorption coefficient in the visible light spectrum (1.8 × 10^5^ cm^−1^ at 450 nm). Rapid, scalable, economically viable and controllable in-air growth of continuous, uniform, polycrystalline Sb_2_S_3_ absorber layers has not yet been accomplished. This could be achieved with chemical spray pyrolysis, a robust chemical method for deposition of thin films. We applied a two-stage process to produce continuous Sb_2_S_3_ optical coatings with uniform thickness. First, amorphous Sb_2_S_3_ layers, likely forming by 3D Volmer–Weber island growth through a molten phase reaction between SbCl_3_ and SC(NH_2_)_2_, were deposited in air on a glass/ITO/TiO_2_ substrate by ultrasonic spraying of methanolic Sb/S 1:3 molar ratio solution at 200–210 °C. Second, we produced polycrystalline uniform films of Sb_2_S_3_ (*E*_g_ 1.8 eV) with a post-deposition thermal treatment of amorphous Sb_2_S_3_ layers in vacuum at 170 °C, <4 × 10^−6^ Torr for 5 minutes. The effects of the deposition temperature, the precursor molar ratio and the thermal treatment temperature on the Sb_2_S_3_ layers were investigated using Raman spectroscopy, X-ray diffraction, scanning electron microscopy, energy dispersive X-ray spectroscopy and UV–vis–NIR spectroscopy. We demonstrated that Sb_2_S_3_ optical coatings with controllable structure, morphology and optical properties can be deposited by ultrasonic spray pyrolysis in air by tuning of the deposition temperature, the Sb/S precursor molar ratio in the spray solution, and the post-deposition treatment temperature.

## Introduction

Antimony sulfide (Sb_2_S_3_) is an environmentally benign material. As Sb and S are abundant elements in the Earth’s crust, enough raw materials can be supplied to manufacture large quantities of Sb_2_S_3_ in the long term. Sb_2_S_3_ can be applied as the inorganic absorber in solar cells due to its direct band gap of ≈1.7 eV [1–2].

Sb_2_S_3_, prepared by a chemical bath deposition (CBD) [3–4], spin coating [5], atomic layer deposition (ALD) [6] or chemical spray pyrolysis (CSP) [7] method, has been applied in extremely thin absorber (ETA) solar cells due to its excellent absorption coefficient in the visible light spectrum (1.8 × 10^5^ cm^−1^ at 450 nm) [1–2]. Improvements in photocurrent density have been sought by utilizing a transparent, nanostructured window layer instead of planar window layers with the ETA Sb_2_S_3_ absorber layer [4,7]. Previous studies show that achieving sufficient repeatability alongside optimization of the component layers, i.e., transparent (structured) window layer, Sb_2_S_3_ absorber layer, and hole transport material layer, and their respective interfaces, is a tremendous undertaking [4].

Attention has surged toward planar heterojunction Sb_2_S_3_ solar cells due to their simpler structure, less intricate production, and enhanced repeatability vs structured solar cells [8]. Planar ≈1.7 eV absorber layers can be applied in semitransparent solar cells as well as in tandem solar cells.

Chemical spray pyrolysis (CSP) is a robust and industrially scalable chemical method for rapid deposition of thin films [9]. Our research group first investigated spray-deposited Sb_2_S_3_ by pneumatically spraying aqueous solutions (tartaric acid added as complexing agent to prevent hydrolysis [10], akin to studies by Rajpure et al. [11]) or methanolic solutions of SbCl_3_. Following, we studied the effect of the Sb/S precursor molar ratio in solution on ultrasonically sprayed Sb_2_S_3_ layers and presented the first planar TiO_2_/Sb_2_S_3_/P3HT solar cells comprising ultrasonically sprayed Sb_2_S_3_ (power conversion efficiency η ≤ 1.9%) [12].

SbCl_3_ and thiourea (SC(NH_2_)_2_) are often used in the field to deposit Sb_2_S_3_ thin films. Spraying the SbCl_3_/SC(NH_2_)_2_ (henceforth Sb/S) 1:6 molar ratio solution at 250 °C in air yielded separate Sb_2_S_3_ grains, which did not cover the TiO_2_ substrate entirely, whereas spraying the Sb/S 1:3 solution yielded an inhomogeneous mix of amorphous and polycrystalline Sb_2_S_3_ [12]. We learned to produce continuous uniform layers of polycrystalline Sb_2_S_3_ by a two-step process on ZnO nanorod/TiO_2_ substrates [7]. In this study, we applied this two-step process, i.e., depositing amorphous Sb_2_S_3_ layers on planar substrates, followed by post-deposition crystallization.

The aim of this study was to produce crystalline, continuous, Sb_2_S_3_ optical coatings with uniform thickness to be applied as a photovoltaic absorber by ultrasonic spraying on planar glass/ITO/TiO_2_ substrates, followed by a post-deposition treatment. To this end, we studied the effect of the deposition temperature (*T*_D_), the molar ratio of precursors SbCl_3_ and thiourea (SC(NH_2_)_2_) in the spray solution, and the post-deposition treatment temperature on the structure, morphology and optical properties of ultrasonically sprayed Sb_2_S_3_ thin films.

## Results and Discussion

Two sequential operations were used to obtain homogeneous Sb_2_S_3_ optical coatings with uniform thickness on planar TiO_2_ substrates. First, we tuned the deposition temperature and molar ratio of Sb/S precursors in spray solution to deposit continuous amorphous Sb_2_S_3_ layers. An intimate contact, which is a prerequisite for high power conversion efficiency in solar cells [13], is formed at the interface between TiO_2_ and Sb_2_S_3_ during deposition of amorphous Sb_2_S_3_ layers. Second, all layers were thermally treated in an inert environment (vacuum, <4 × 10^−6^ Torr) to induce crystallization, without oxidation.

Preliminary experiments at deposition temperatures lower than 182 °C (decomposition of SC(NH_2_)_2_ [14–15]) yielded inhomogeneous red-brown layers. Furthermore, in our previous paper, 250 °C was found to be too high a deposition temperature to obtain sufficient coverage of TiO_2_ substrate by polycrystalline Sb_2_S_3_ thin films, despite the suitable band gap of 1.6 eV and high phase purity [12]. Restricted to deposition temperatures in the range 182–250 °C, we sprayed Sb/S 1:3 and 1:6 molar ratio precursor solutions at *T*_D_ = 200, 210, and 220 °C. We varied the aforementioned parameters to attain the conditions to deposit dense and homogeneous layers of amorphous Sb_2_S_3_, which we then crystallized by a post-deposition thermal treatment.

Based on the scanning electron microscopy (SEM) images, preliminary experiments revealed that spraying Sb/S 1:6 solutions consistently yielded twice thinner layers compared to layers deposited from Sb/S 1:3 solutions. Sb_2_S_3_ layers of comparable thickness were deposited by spraying Sb/S 1:6 solutions for 40 minutes and Sb/S 1:3 solutions for 20 minutes.

The samples are named in the text as follows: A-B-C, where A is the S/Sb molar ratio in solution, B is the deposition temperature, and C is the specification of the treatment. [Sb/S molar ratio in solution: “3” for Sb/S 1:3 or “6” for Sb/S 1:6]-[deposition temperature: “200”, “210” or “220” (°C)]-[treatment: “As-dep.” for as-deposited and “170”, “200” or “250” (°C) for samples thermally treated in vacuum].

The samples in which Sb_2_S_3_ layers were deposited from either Sb/S 1:3 or 1:6 solution at *T*_D_ = 200 °C, followed by thermal treatment in vacuum at 200 °C (3-200-200, 6-200-200), contain no Sb_2_S_3_, as it likely volatilized completely during the vacuum thermal treatment. Likewise, treating the Sb_2_S_3_ layers at temperatures higher than 200 °C caused Sb_2_S_3_ to completely volatilize during treatment. Photographs of the samples (Figure S1) and the description of the vapor pressure calculations (Comment S1) are provided in the Supporting Information File 1. Consequently, only as-deposited samples and samples thermally treated in vacuum at 170 °C and 200 °C are eligible for discussion.

### Structure of as-deposited and thermally treated Sb_2_S_3_ layers

**Figure 1:**
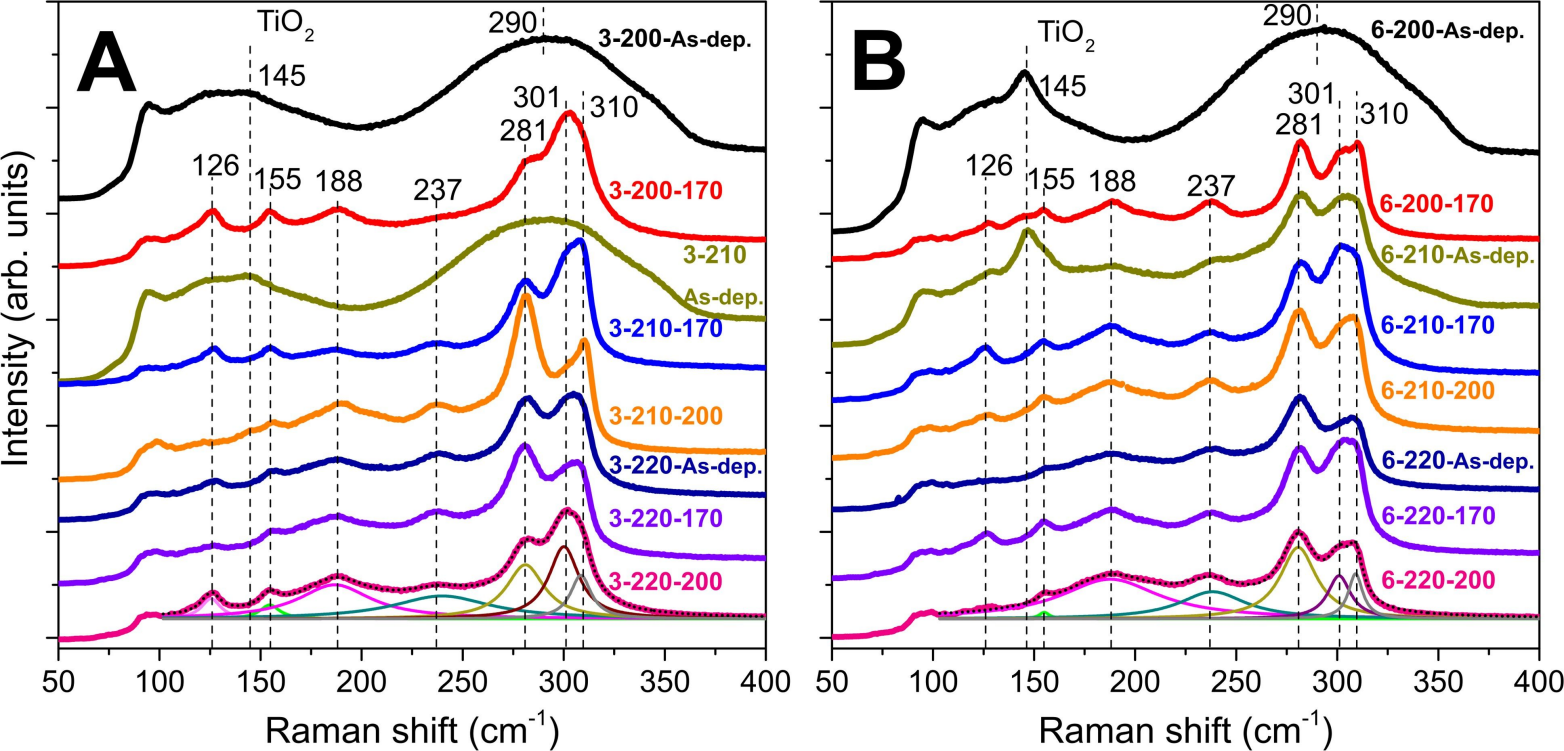
Raman spectra (shifted for visibility) of the as-deposited and thermally treated Sb_2_S_3_ films deposited from Sb/S 1:3 (A) or 1:6 (B) solution at 200, 210, 220 °C. Examples of deconvoluted fitted band curves are presented for the lowermost spectra. Sample names in figures: [S/Sb molar ratio in solution]-[deposition temperature]-[thermal treatment temperature].

Raman spectroscopy provides quantitative and qualitative information on the vibrational modes in solids. The wide Raman band centered at 290 cm^−1^ [12,16] associated with metastibnite, i.e., amorphous Sb_2_S_3_, is characteristic of as-deposited orange colored (photograph in Supporting Information File 1, Figure S1) samples (3-200-As-dep., 3-210-As-dep., Figure 1A; 6-200-As-dep., Figure 1B). The band centered at 145 cm^−1^ is a low frequency *E*_g_ vibrational mode of anatase-TiO_2_ [17], which is observed due to the laser beam penetrating to the substrate [12,16] through the discontinuous Sb_2_S_3_ layers. The TiO_2_ vibrational band is absent in spectra of Sb_2_S_3_ layers containing less pinholes, as the signal is captured only from Sb_2_S_3_.

The narrower bands, attributed to orthorhombic Sb_2_S_3_ [16,18–20], are present in the spectra of as-deposited and thermally treated lustrous gray (photograph in Supporting Information File 1, Figure S1) samples (3-200-170, 3-210-170, 3-210-200, 3-220-As-dep., 3-220-170, 3-220-200, Figure 1A; 6-200-170, 6-210-As-dep., 6-210-170, 6-210-200, 6-220-As-dep., 6-220-170, 6-220-200, Figure 1B; photograph in Supporting Information File 1, Figure S1). According to group theory, orthorhombic Sb_2_S_3_ has 30 predicted Raman active modes: Γ_Raman_ = 10*A*_g_ + 5*B*_1g_ + 10*B*_2g_ + 5*B*_3g_ [18,20]. The Raman spectra were deconvoluted using Lorentzian fitting into vibrational bands of Sb_2_S_3_ based on the literature [12,16,21–22]. The centers of the bands of Sb_2_S_3_ in the deconvoluted Raman spectra (Table 1, symmetries taken from [20–21]) are similar to values reported in our previous studies [7,12]. Band centers, relative single peak intensities and full widths at half maximum (FWHM) of the narrow bands centered at 281, 301 and 310 cm^−1^ can be respectively found in Tables S1, S2, and S3 of Supporting Information File 1.

**Table 1:** Raman band centers and assigned active modes for the studied Sb_2_S_3_ layers.

Center of Raman band, cm^−1^			Symmetry		Vibrational mode, [21–23]
This study	Ref. [21]	Ref. [20]	Ref. [21]	Ref. [20]	

126	125	129	*A*_g_	*A*_g_	lattice mode
155	156	158	*A*_g_	*A*_g_/*B*_2g_	lattice mode
188	189	186	*B*_1g_	*B*_1g_	antisym. S–Sb–S bending
237	237	239	*B*_1g_	*B*_1g_/*B*_3g_	symmetric S–Sb–S bending
281	281	282	*A*_g_	*A*_g_/*B*_2g_	antisym. S–Sb–S stretching
301	300	299	*A*_g_	*A*_g_/*B*_2g_	antisym. S–Sb–S stretching
310	310	312	*A*_g_	*A*_g_/*B*_2g_	symmetric S–Sb–S stretching

The FWHM of the vibrational band centered at 281 cm^−1^ narrows from ≈24 cm^−1^ to 21–23 cm^−1^ after vacuum thermal treatment of the samples deposited at 210–220 °C from both Sb/S 1:3 and Sb/S 1:6 solutions (3-210-170, 3-220-170, 6-210-170 and 6-220-170) at 170 °C (3-210-170, 3-220-170, 6-210-170 and 6-220-170) and narrows by 5 cm^−1^ at most after vacuum thermal treatment at 200 °C (3-210-200). The narrowing of the Raman bands due to thermal treatment leads us to suppose that crystallization continues during the vacuum thermal treatment and proceeds further at higher thermal treatment temperatures [16]. The vibrational bands corresponding to Sb_2_O_3_ were not detected by Raman spectroscopy in any of the studied glass/ITO/TiO_2_/Sb_2_S_3_ samples.

**Figure 2:**
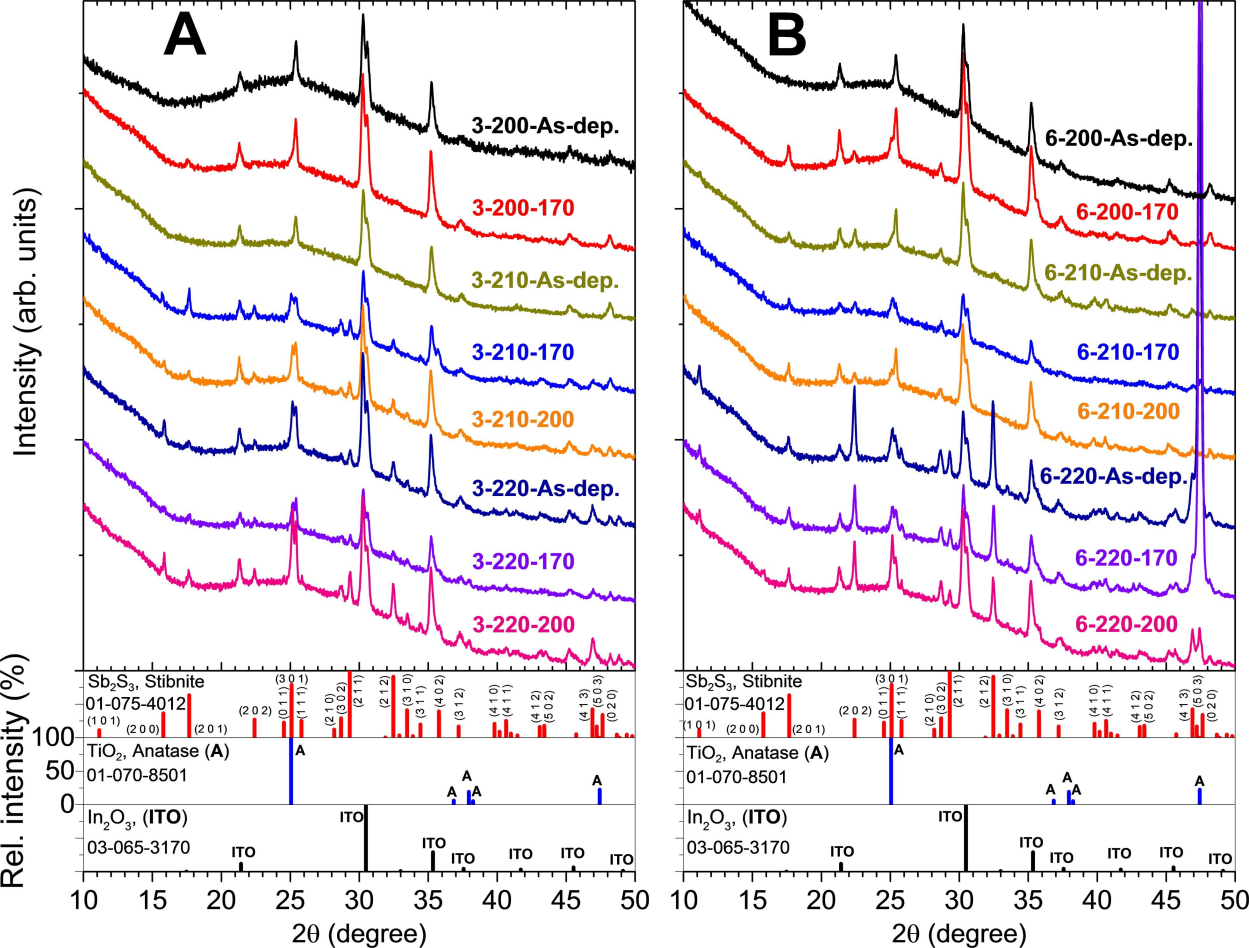
XRD patterns (shifted for visibility) of as-deposited and vacuum treated (170 °C or 200 °C, 5 minutes) Sb_2_S_3_ layers deposited on glass/ITO/TiO_2_ substrate from Sb/S 1:3 (A) or 1:6 (B) solution at *T*_s_ = 200, 210, 220 °C. Sample names in figures: [S/Sb molar ratio in solution]-[deposition temperature]-[thermal treatment temperature].

X-ray diffraction (XRD) provides qualitative information on the phase composition and crystal structure. XRD patterns of reference glass/ITO/TiO_2_ samples and samples containing XRD-amorphous Sb_2_S_3_ (3-200-As-dep., 3-210-As-dep., Figure 2A; 6-200-As-dep., Figure 2B) show only diffraction peaks corresponding to cubic In_2_O_3_ (2θ = 21.3°, 30.4°, 35.3°, 37.4°, 41.4°, 45.3°, ICDD PDF 03-065-3170) and anatase-TiO_2_ (25.3°, 48.2°, ICDD PDF 00-016-0617). The diffraction peaks of orthorhombic Sb_2_S_3_ (ICDD PDF 01-075-4012), space group *Pnma* (*D*_2h_^16^) [20,24–25]*,* appear in XRD patterns of lustrous gray as-deposited and thermally treated Sb_2_S_3_ samples (3-200-170, 3-210-170, 3-210-200, 3-220-As-dep., 3-220-170, 3-220-200, Figure 2A; 6-200-170, 6-210-As-dep., 6-210-170, 6-210-200, 6-220-As-dep., 6-220-170, 6-220-200, Figure 2B). The 2θ angles of observed Sb_2_S_3_ diffraction peaks and corresponding crystal plane indices are presented in Supporting Information File 1, Table S4. Experimentally determined mean lattice constants *a*, *b* and *c* of Sb_2_S_3_ are 11.25 ± 0.07 Å, 3.810 ± 0.025 Å and 11.16 ± 0.07 Å, respectively. Our experimentally determined mean unit cell volume (479 ± 4 Å^3^) lies between the experimentally determined volume (486.7 Å^3^) and the theoretically determined volume (470.5 Å^3^) calculated from orthorhombic Sb_2_S_3_ powder (>99.99 wt %) data presented by Ibáñez et al. [20].

Sb_2_S_3_ layers deposited from Sb/S 1:6 solution at 210 °C (6-210-As-dep., Figure 2B) are polycrystalline, whereas layers deposited from Sb/S 1:3 solution (3-210-As-dep., Figure 2A) are XRD-amorphous. Sb_2_S_3_ layers deposited at 220 °C from both Sb/S 1:3 (3-220-As-dep., Figure 2A) and 1:6 (6-220-As-dep., Figure 2B) solution are polycrystalline. Several diffraction peaks corresponding to orthorhombic Sb_2_S_3_ were detected in these samples. No additional phases were detected by XRD in any studied samples. The presence or absence of amorphous Sb_2_O_3_ as a minor phase in the Sb_2_S_3_ layers, as it is difficult to ascertain by Raman or XRD analyses, has not been conclusively demonstrated.

The diffraction peak of the (2 0 0)/(0 0 2) plane of Sb_2_S_3_ is absent in most samples deposited from Sb/S 1:6 solution. Conversely, the diffraction peak of the (1 0 1) plane of Sb_2_S_3_ is absent in all samples deposited from Sb/S 1:3 solution. Sb_2_S_3_ crystallites in most of our samples have no preferred orientation. Only crystallites in as-deposited and vacuum treated (170 °C) samples deposited from Sb/S 1:6 solution (6-220-As-dep., 6-220-170, Figure 2B) show a preferred orientation parallel to the substrate surface along the (0 2 0) plane normal of Sb_2_S_3_. Interestingly, this preferred orientation of crystallites does not extend to the sample with Sb_2_S_3_ deposited in the same conditions, but thermally treated in vacuum at 200 °C (6-220-200, Figure 2B).

The larger crystallite size is a boon to the power conversion efficiency of all solar absorber materials because decreasing the amount of grain boundaries likely increases charge carrier mobility [26]. The crystallite sizes of as-deposited and thermally treated Sb_2_S_3_ layers are presented in Table 2. The effect of the deposition temperature is observed in Sb/S 1:3 Sb_2_S_3_ layers, as the crystallite size increases after vacuum annealing at 170 °C from 19 ± 8 nm to 100 ± 23 nm by raising *T*_D_ from 200 to 220 °C. The crystallite size in Sb/S 1:6 Sb_2_S_3_ layers (42 ± 15 nm) does not change significantly with *T*_D_ or vacuum treatment. Furthermore, vacuum treatment at 200 °C vs 170 °C does not substantially affect the crystallite size of Sb_2_S_3_ layers.

**Table 2:** Crystallite size (*D*) of as-deposited and vacuum treated Sb_2_S_3_ thin films. The crystallite size was calculated by the Scherrer equation from the (2 0 2) diffraction peak of as-deposited and vacuum treated (170 °C, 200 °C, 5 minutes) Sb_2_S_3_ thin films deposited on glass/ITO/TiO_2_ substrates from Sb/S 1:3 and 1:6 precursor solution at *T*_D_ = 200, 210, 220 °C.

	*D*, nm					
Sb/S in sol.	1:3			1:6		
*T*_D_, °C	200	210	220	200	210	220

as-dep.	amorph.	amorph.	33 ± 10	amorph.	39 ± 4	47 ± 1
vac. 170 °C	19 ± 8	38 ± 6	100 ± 23	37 ± 8	35 ± 4	49 ± 3
vac. 200 °C	no layer^a^	32 ± 8	67 ± 12	no layer^a^	45 ± 6	52 ± 3

^a^No Sb_2_S_3_ was detected by XRD or Raman.

In comparison, the largest crystallites in Sb_2_S_3_ layers grown on TiO_2_ substrates via CBD and annealed at 270 °C in N_2_ for 30 min oriented along the (2 0 0) plane parallel to the substrate were 74 nm in size [16]. The crystallites oriented along the (2 0 1) plane were 24 nm in size in Sb_2_S_3_ layers grown on SnO_2_/F (FTO) coated glass substrates via thermal evaporation [27]. The crystallite size was 52 nm along the (3 0 1) plane in Sb_2_S_3_ layers grown on glass substrates at 250 °C via spray pyrolysis [28], similar to the crystallite size in some of our samples. We conclude that the mean crystallite size in our Sb_2_S_3_ layers is in the general range of values obtained in the literature using both chemical and physical methods.

### Morphology of as-deposited and thermally treated Sb_2_S_3_ layers

#### Influence of deposition temperature on morphology of Sb_2_S_3_ layers

**Figure 3:**
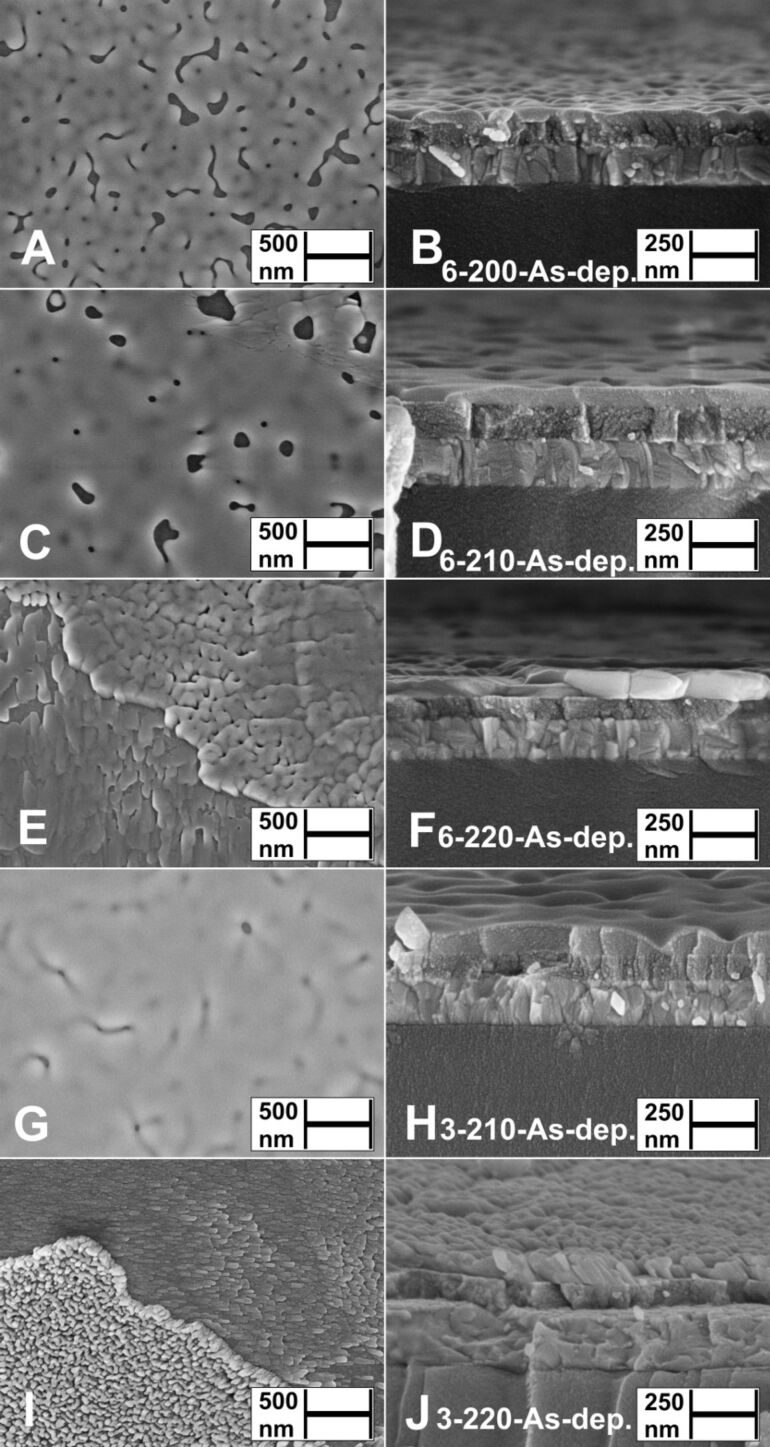
Surface and cross-sectional views by SEM study of as-deposited Sb_2_S_3_ layers deposited from Sb/S 1:6 solution at *T*_D_ = 200 °C (A, B), 210 °C (C, D) or 220 °C (E, F) and from Sb/S 1:3 solution at *T*_D_ = 210 °C (G, H) or 220 °C (I, J) on glass/ITO/TiO_2_ substrate. Sample names in figures: [S/Sb molar ratio in solution]-[deposition temperature]-[as-deposited].

The aim of this study was to obtain uniform Sb_2_S_3_ layers, which continuously coat the TiO_2_ substrate. According to SEM surface studies, layers deposited from both Sb/S 1:3 and Sb/S 1:6 solutions at 200 and 210 °C (3-200-As-dep., 3-210-As-dep., Figure 3G,H, Supporting Information File 1, Figure S2A,B, Figure S3A,B; 6-200-As-dep., Figure 3A,B; 6-210-As-dep., Figure 3C,D) cover the substrate almost entirely. Grain boundaries and larger clusters of grains have formed in layers deposited from Sb/S 1:6 solutions for 40 minutes at 210 °C (6-210-As-dep., Figure 3C,D, Figure S5C,D). Cap-shaped islands (Ø 70 nm) in Sb_2_S_3_ layers deposited from Sb/S 1:6 solution at *T*_D_ = 210 °C for 20 minutes (Figure S4A,B), have grown (Ø 100 nm) and coalesced further after 40 minutes of deposition at 200–210 °C (6-200-As-dep., Figure 3A,B, Figure S5A,B; 6-210-As-dep., Figure 3C,D, Figure S5C,D, Figure S6A,B), thereby covering the TiO_2_ substrate to a greater extent. The layers deposited from Sb/S 1:6 solution at 220 °C for 40 minutes (6-220-As-dep., Figure 3E,F, Figure S5E,F) consist of various agglomerates, separated by pinholes, and grains flowing randomly along the partially exposed TiO_2_ substrate (lower left, Figure 3E).

Increasing the deposition temperature from 210 to 220 °C significantly transforms the surface morphology in Sb/S 1:3 layers, as instead of the planar grains (3-210-As-dep., Figure 3G,H) domains of elongated rod-shaped grains (length ≈ 100 nm) appear either upright or sideways on the substrate (3-220-As-dep., Figure 3I,J, Figure S3C,D). Rod-shaped Sb_2_S_3_ grains were able to grow due to the nature of the material as well as due to complex interactions between the substrate and the turbulence of the spray during deposition [29].

Increasing the sulfur precursor concentration in the spray solution from Sb/S 1:3 to 1:6 (and deposition time from 20 to 40 minutes) yields Sb_2_S_3_ layers consisting of agglomerated grains (6-220-As-dep., Figure 3E,F). As the deposition time was simultaneously increased from 20 to 40 minutes, it is uncertain whether the morphology of the Sb_2_S_3_ layers is affected more by the Sb/S molar ratio in solution or by the deposition time. Sb_2_S_3_ tends to yield different morphologies in similar deposition conditions, possibly due to liquid phase reactions between molten-boiling SbCl_3_ (mp 73.4 °C, bp 223.5 °C [30]) and molten thiourea (TU, mp 182 °C [14–15]) catalyzed by the highly active surface of the TiO_2_ substrate [31].

We have consistently observed twice slower growth of Sb_2_S_3_ by spraying solutions with Sb/S 1:6 (Supporting Information File 1, Figure S4A,B) vs Sb/S 1:3 (Figure 3G,H) molar ratio at 200–220 °C. We speculate that doubling the concentration of TU could sterically inhibit the formation of solid Sb_2_S_3_ nuclei on the surface of the active TiO_2_ substrate due to more intense bubbling of volatile TU decomposition products (CS_2_, NH_3_, HCN, COS, SO_2_, HCl, HNCS at 200–220 °C in air based on decomposition studies of pure TU [14], Cu(TU)_3_Cl [32], Zn(TU)_2_Cl_2_ [33], and Sn(TU)_2_Cl_2_ [34]) in the surrounding liquid phase.

In summary, the most uniform and continuous Sb_2_S_3_ thin films were deposited from Sb/S 1:3 solution at 200–210 °C.

#### Influence of vacuum treatment temperature on morphology of Sb_2_S_3_ layers

**Figure 4:**
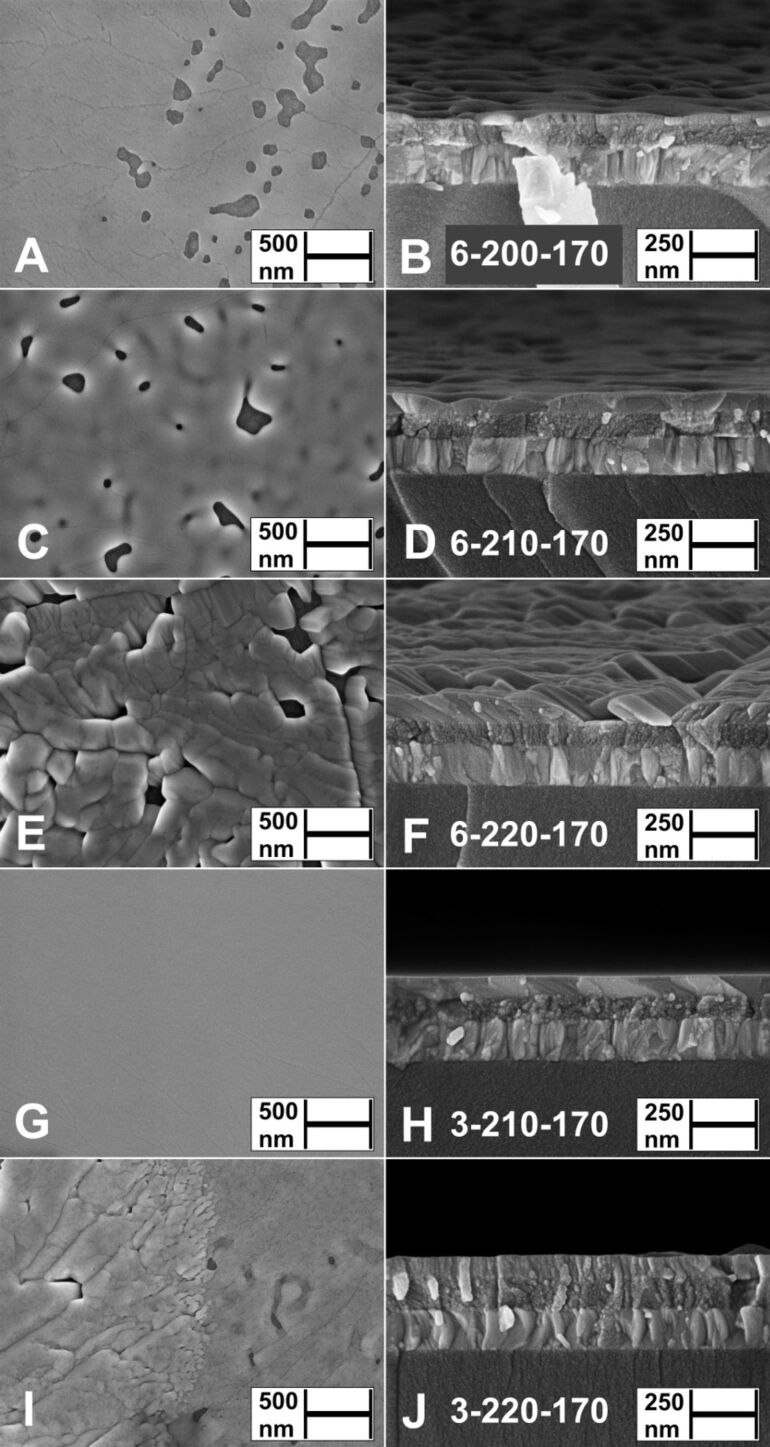
Surface and cross-sectional views by SEM study of thermally treated (170 °C, 5 minutes) Sb_2_S_3_ layers deposited from Sb/S 1:6 solution at *T*_D_ = 200 °C (A, B), 210 °C (C, D) or 220 °C (E, F) and from Sb/S 1:3 solution at *T*_D_ = 210 °C (G, H) or 220 °C (I, J) on glass/ITO/TiO_2_ substrates. Sample names in figures: [S/Sb molar ratio in solution]-[deposition temperature]-[thermal treatment temperature].

The thermal treatment of X-ray amorphous Sb_2_S_3_ layers (6-200-As-dep., Figure 3A,B; 3-200-As-dep.; 3-210-As-dep., Figure 3G,H, Supporting Information File 1, Figure S2A,B) in vacuum at 170 °C for 5 minutes yields enhanced substrate coverage at the expense of decreased layer thickness due to coalescence of grains and film formation (6-200-170, Figure 4A,B; 3-200-170, Figure 4G,H; 3-210-170, Figure 4I,J). Complete substrate coverage is observed in the Sb_2_S_3_ layers deposited at 210 °C from Sb/S 1:3 solution as coalescence is facilitated during treatment in vacuum at 170 °C due to the near-continuous coverage of the TiO_2_ substrate in the as-deposited layers (3-210-170, Figure 4G,H, Figure S2C,D, Figure S7A,B).

Planar grain agglomerates in thermally treated Sb_2_S_3_ layers (3-210-170, Figure 4G,H, Supporting Information File 1, Figure S7A,B; 6-200-170, Figure 4A,B, Figure S9A,B; 6-210-170, Figure 4C,D, Figure S9C,D) range from 100 nm to over 10 µm in size. These agglomerates, consisting of smaller grains separated by ridges, resemble the surface morphology of 300 nm thick polycrystalline Sb_2_S_3_ films grown via thermal evaporation and annealed for 10 min at 300 °C in N_2_ [35], and that of metal halide perovskites obtained by Volmer–Weber growth via hot casting [36]. The layers deposited at 220 °C from both Sb/S 1:3 and Sb/S 1:6 solutions, and thermally treated at 170 °C, consist of numerous grains and pinholes (3-220-170, Figure 4I,J; 6-220-170, Figure 4E,F).

**Figure 5:**
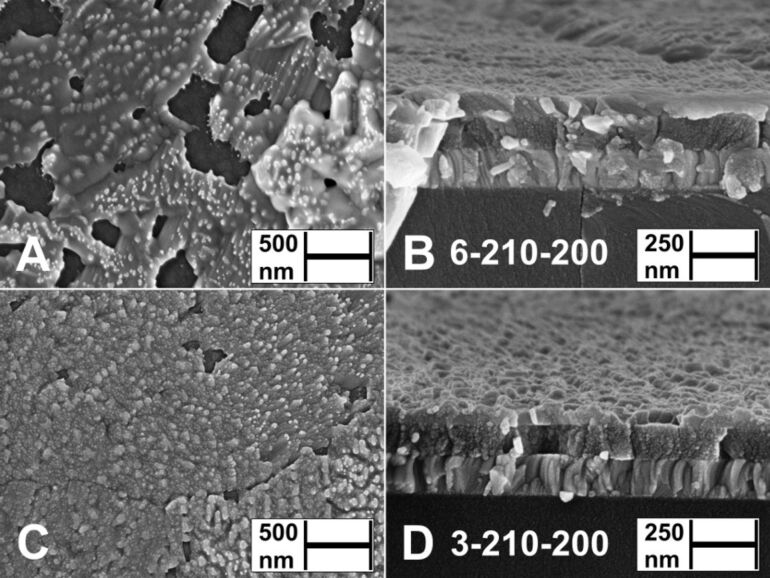
Surface and cross-sectional views by SEM study of vacuum treated (200 °C, 5 minutes) Sb_2_S_3_ layers deposited from Sb/S 1:6 solution (A, B) and from Sb/S 1:3 solution (C, D) at *T*_D_ = 210 °C on glass/ITO/TiO_2_ substrates. Sample names in figures: [S/Sb molar ratio in solution]-[deposition temperature]-[thermal treatment temperature].

Sb_2_S_3_ layers deposited at 210 °C from both Sb/S 1:3 and Sb/S 1:6 solutions, and thermally treated in vacuum at 200 °C (3-210-200, Figure 5A,B, Supporting Information File 1, Figure S8A,C,E; 6-210-200, Figure 5C,D, Figure S8B,D,F), are porous, inhomogeneous and ≈20 nm thinner (Table 3) vs the uniform in thickness layers after treatment at 170 °C (3-210-170, Figure 4I,J; 6-210-170, Figure 4C,D).

**Table 3:** Thicknesses of Sb_2_S_3_ layers estimated from SEM images.

	Sb_2_S_3_ layer thickness, nm					
	Sb/S 1:3 in sol., 20 min dep.			Sb/S 1:6 in sol., 40 min dep.		

*T*_D_, °C	200	210	220	200	210	220
as-dep.	70–90	80–100	60/150^a^	50–70	60/400^a^	40/400^a^
vac., 170 °C	70–90	70–90	80/150^a^	30–40	60/400^a^	40/400^a^
vac., 200 °C	no layer^b^	60–70	N/A	no layer^b^	60–70	N/A

^a^Thickness of formations shown in the Supporting Information File 1 in Figures S5, S7, S8 and S9. ^b^No Sb_2_S_3_ was detected by XRD or Raman.

The decreasing layer thickness indicates that approximately a quarter of Sb_2_S_3_ by volume has either evaporated or sublimated, i.e., volatilized. Incongruent evaporation, i.e., depletion of sulfur in Sb_2_S_3_ during evaporation, may cause the change in Sb_2_S_3_ layer morphology, as volatilization of the planar regions around the nucleating islands has been reported during thermal treatment of both Sb_2_Se_3_ layers grown via thermal evaporation [37] and oxide containing Sb_2_S_3_ layers grown via CBD [16].

The calculated vapor pressure of Sb_2_S_3_ is ≈2 × 10^−10^ Torr at 170 °C, 7 × 10^−9^ Torr at 200 °C and 9 × 10^−7^ Torr at 250 °C [38], whereas the dynamic system pressure is ≈4 × 10^−6^ Torr. The calculated partial pressure of Sb_2_S_3_ is ≈0.0050% at 170 °C, 0.18% at 200 °C and 23% at 250 °C (Comment S1 in Supporting Information File 1). The loss of a quarter of the Sb_2_S_3_ layer thickness in samples that were vacuum annealed at 200 vs 170 °C (Table 3) correlates with the exponential increase in Sb_2_S_3_ vapor pressure in the 170–250 °C range.

In conclusion, the most uniform and continuous Sb_2_S_3_ thin films were produced by vacuum treatment at 170 °C for 5 min of Sb_2_S_3_ layers deposited from Sb/S 1:3 solution at 200–210 °C.

### Elemental composition of as-deposited and thermally treated Sb_2_S_3_ layers

The elemental composition of Sb_2_S_3_ in as-deposited and thermally treated glass/ITO/TiO_2_/Sb_2_S_3_ samples was determined using energy dispersive X-ray spectroscopy (EDX). The EDX results of studied Sb_2_S_3_ layers in terms of S to Sb atomic ratio (S/Sb) are presented in Table 4. S/Sb in both as-deposited and vacuum annealed polycrystalline Sb_2_S_3_ layers deposited at *T*_D_ = 220 °C is close to the stoichiometric value of 1.5 of Sb_2_S_3_, whereas the S/Sb ratio of as-deposited and thermally treated Sb_2_S_3_ layers (Sb/S 1:3 in solution, *T*_D_ 200–210 °C, 3-200-As-dep., 3-210-As-dep., 3-200-170, 3-210-170) is ≈1.3. S/Sb is ≈1.5–1.6 in layers deposited from Sb/S 1:6 solution at 200–220 °C.

**Table 4:** S/Sb atomic ratio of as-deposited and thermally treated Sb_2_S_3_ layers calculated from EDX data.

	S/Sb in layer					
Sb/S in sol.	1:3			1:6		
*T*_D_, °C	200	210	220	200	210	220

as-dep.	1.3	1.3	1.5	1.6	1.5	1.5
vac., 170 °C	1.3	1.3	1.5	1.6	1.6	1.5
vac., 200 °C	N/A	1.4	N/A	N/A	1.5	N/A

We note that interpretation of EDX spectra of very thin layers is difficult. Most of our Sb_2_S_3_ layers are thinner than 100 nm, which could explain the divergence in the elemental composition of our Sb_2_S_3_ layers. Therefore, future studies by more surface sensitive methods are required. Overall, S/Sb in most studied samples approximates the stoichiometric value of 1.5 of Sb_2_S_3_.

Oxygen could not be quantified by EDX due to the thin layers and high concentration of O in the glass/ITO/TiO_2_ substrate. In addition, C and Cl levels were below the detection limit of the used EDX setup in all studied Sb_2_S_3_ layers, meaning most C and Cl species exit the growing Sb_2_S_3_ layer during deposition in open environment (Supporting Information File 1, Figure S11). We believe that this reinforces our claim that formation of Sb_2_S_3_ proceeds through a molten phase reaction between SbCl_3_ and TU, where the denser (4562 kg/m^3^ [39]) Sb_2_S_3_ precipitates and nucleates, while the remainder of the volatile compounds (SbCl_3_, and various decomposition products of TU) exit the system [14–15,38,40].

### Growth mechanism of Sb_2_S_3_ layers by spray pyrolysis

The three most common growth mechanisms of solids can be described by the following equations [41]:

[1] $$\sigma_{\text{SG}}>\sigma_{\text{LG}}+\sigma_{\text{SL}}$$

[2] $$\sigma_{\text{SG}}<\sigma_{\text{LG}}+\sigma_{\text{SL}}$$

[3] $$\sigma_{\text{SG}}\approx \sigma_{\text{LG}}+\sigma_{\text{SL}}$$

Where σ_SG_ is the surface free energy of the substrate–gas interface (TiO_2_–air), σ_LG_ is the surface free energy of the layer–gas interface (Sb_2_S_3_–air) and σ_SL_ is the surface free energy of the substrate–layer interface (TiO_2_–Sb_2_S_3_). The surface free energy (σ) is the driving force of fluids and solids to seek a condition of minimum energy by contracting interfacial surface area [41]. Separate 3D islands grow if Equation 1 is valid, a.k.a. Volmer–Weber growth; 2D layer-by-layer growth occurs if Equation 2 is valid, a.k.a. Frank–Van der Merwe growth; combined 2D layer-by layer and 3D island growth occurs if Equation 3 is valid, a.k.a. Stranski–Krastanov growth [36,41–43].

Furthermore, SEM surface studies show cap-shaped islands indicative of Volmer–Weber growth in Sb_2_S_3_ layers deposited on Si/SiO_2_ alternative substrates by ultrasonic spraying (Supporting Information File 1, Figure S10A,B). Metastibnite-Sb_2_S_3_ forms when formation of stibnite-Sb_2_S_3_ is halted by insufficient reaction time and energy [44–46]. Volmer–Weber island growth of amorphous Sb_2_S_3_ (and in some cases leaf-like grains of polycrystalline Sb_2_S_3_) have been observed in Sb_2_S_3_ layers grown by chemical bath deposition on glass [47–48], In_2_O_3_/Sn (ITO) [49], planar TiO_2_ [16] and TiO_2_ nanotube arrays [50], by sequential deposition [51] and spin coating [8,52] on planar TiO_2_, by photochemical deposition on mesoporous TiO_2_ [53], by thermal evaporation on planar CdS [27] and planar TiO_2_ [54]. Supported by these numerous observations, we consider the Volmer–Weber growth characteristic of Sb_2_S_3_, given that the substrate and deposition conditions are met. Indeed, metastibnite, the naturally occurring mineral form of amorphous Sb_2_S_3_, has the botryoidal characteristic, preferentially forming globular clusters [55]. We have also observed 3D growth of extremely thin TiO_2_ layers by spray pyrolysis [56]. Therefore, 3D island growth may partially be imposed by the use of the spray pyrolysis method as well.

**Figure 6:**
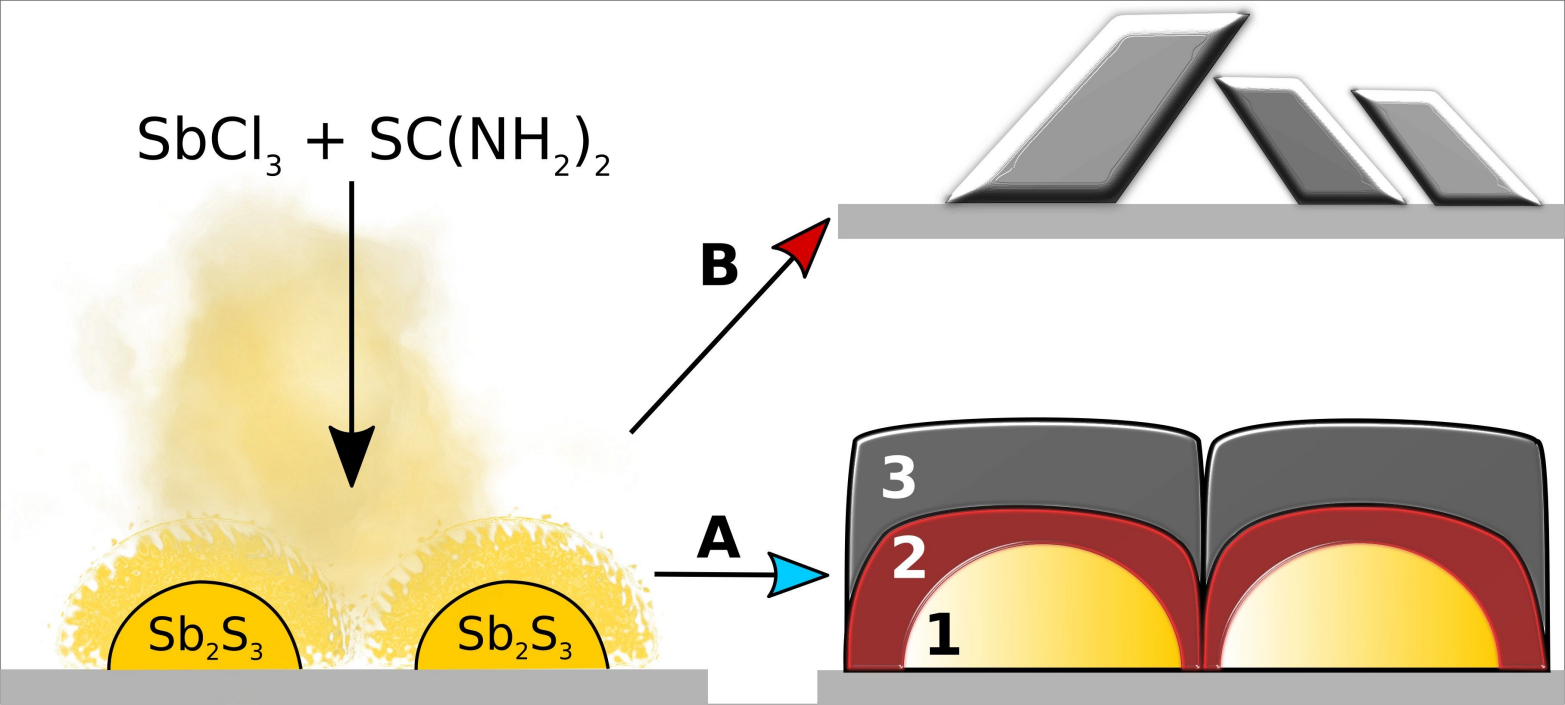
Proposed growth mechanism paths of Sb_2_S_3_ by Volmer–Weber growth during ultrasonic spraying of methanolic solution of SbCl_3_–SC(NH_2_)_2_ in excess of sulfur precursor in aerosol. Amorphous Sb_2_S_3_ nucleates after precipitation from a molten SbCl_3_–SC(NH_2_)_2_ mixture: A – Amorphous Sb_2_S_3_ islands nucleate on the rigid TiO_2_ substrate and grow by 3D Volmer–Weber growth, surrounded by a protective bubbling liquid film of volatile SbCl_3_ and TU decomposition products (1), eventually interconnecting by coalescence of sufficiently large islands to minimize Sb_2_S_3_–air interfacial free surface energy (2), and form grain boundaries during crystallization in vacuum or inert environment (3). B – Sb_2_S_3_ crystallizes into separate grains if either the deposition temperature, the deposition time or the excess of TU in Sb/S precursor molar ratio exceed a critical value before or during process A, i.e., the energetic threshold for crystallization is surpassed.

Based on the above observations, the morphology and crystallinity of as-deposited layers seems to determine the nature of Sb_2_S_3_ layer morphology as formed during vacuum thermal treatment. Our proposed growth mechanism of Sb_2_S_3_ by ultrasonic spraying in air is illustrated in Figure 6.

### Optical properties of as-deposited and thermally treated Sb_2_S_3_ layers

The absorption coefficient (α) and band gap (*E*_g_) values of Sb_2_S_3_ in both as-deposited and thermally treated glass/ITO/TiO_2_/Sb_2_S_3_ samples were determined using an approximated Sb_2_S_3_ layer thickness of 100 nm derived from SEM images (Table 3). The absorption coefficient α was determined as

[4] $$\alpha =d^{-1}\ln\left[\left(1-R\right)T^{-1}\right]\;,$$

where *d* is the layer thickness, *R* is the total reflectance, included to compensate for thin film interference, and *T* is the total transmittance.

The band gap of Sb_2_S_3_ layers was determined by plotting (α*h*ν)^1/r^ vs *h*ν, where *h* is the Planck constant, ν is the frequency and *r* = 1/2 is the exponent corresponding to the assumed direct optical transition [57]. Extrapolating the linear region of this curve to the *h*ν-axis yields the optical band gap. Thin film interference could not be completely removed by accounting for reflectance in α calculations. Thus, the absolute values of α may deviate from the expected values with the uncertainty introduced by using a constant layer thickness in calculations.

**Figure 7:**
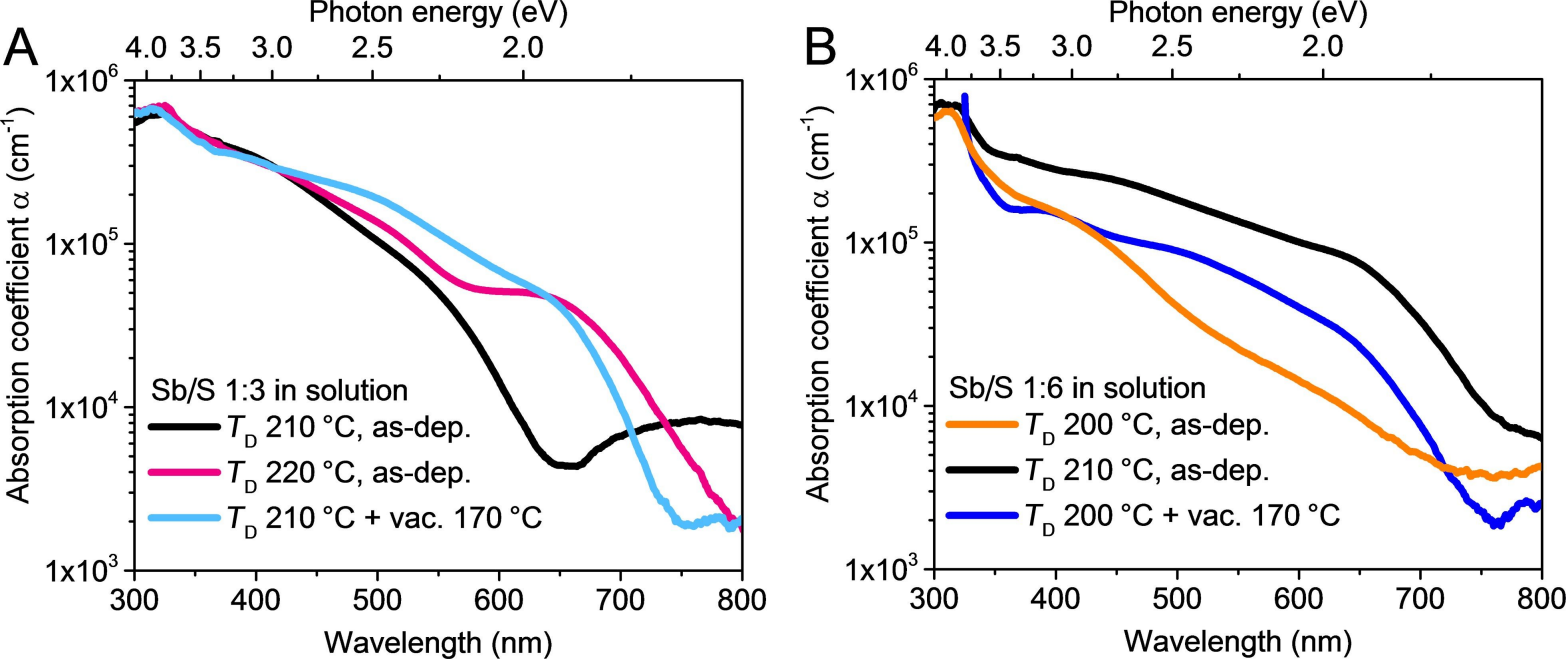
Absorption coefficient (α) vs wavelength of glass/ITO/TiO_2_/Sb_2_S_3_ samples incorporating as-deposited and vacuum treated (170 °C, 5 minutes) Sb_2_S_3_ layers. The α of glass/ITO/TiO_2_ substrates is not shown as it is negligible at the presented wavelengths. Sb_2_S_3_ layers were deposited from Sb/S 1:3 solution at 210 °C, 220 °C (A) and from Sb/S 1:6 solution at 200 °C and 210 °C (B).

The α vs wavelength plots of samples, which contain as-deposited or vacuum-treated Sb_2_S_3_ layers deposited from Sb/S 1:3 solution, are shown in Figure 7A. Likewise, α vs wavelength plots of Sb/S 1:6 samples are shown in Figure 7B. The α in samples containing amorphous Sb_2_S_3_ increases steadily from 10^3^–10^4^ cm^−1^ at 600–800 nm to 10^5^ cm^−1^ at around 400 nm. The α increases significantly faster in samples containing as-grown crystalline Sb_2_S_3_ or vacuum crystallized Sb_2_S_3_. The value of α surges by an order of magnitude from around 10^4^ cm^−1^ to 10^5^ cm^−1^ as the wavelength decreases from 750 nm to 650 nm due to the onset of absorption in crystalline Sb_2_S_3_. At shorter wavelengths beyond the absorption edge, α increases at a slower rate, from around 10^5^ cm^−1^ at 650 nm to more than 5 × 10^5^ cm^−1^ at 300 nm. The optical absorption results are in agreement with XRD, which shows that these samples (3-220**-**As-dep., 3-210-170, 6-210-As-dep. and 6-200-170) contain orthorhombic Sb_2_S_3_ (Figure 2A,B). Comparing the α spectra of samples containing amorphous and crystalline Sb_2_S_3_ further confirms that the Sb_2_S_3_ layers deposited from Sb/S 1:3 solution at 200–210 °C, and from Sb/S 1:6 solution at 200 °C, are indeed amorphous. Namely, α is an order of magnitude smaller at around 600 nm in samples containing amorphous Sb_2_S_3_ layers (3-210-As-dep. and 6-200-As-dep.).

The experimentally determined *E*_g_ are ≈2.7 and 1.8 eV for amorphous and polycrystalline Sb_2_S_3_, respectively (Table 5, Tauc plots in Supporting Information File 1, Figure S12). In comparison, *E*_g_ of amorphous CBD-Sb_2_S_3_ on glass substrates is ≈2.5 eV [58] and *E*_g_ of polycrystalline Sb_2_S_3_ prepared by physical and chemical methods is commonly reported as 1.6–1.8 eV [1,22,58–60]. As such, we find the *E*_g_ of our polycrystalline Sb_2_S_3_ layers lies satisfactorily in the range of published values.

**Table 5:** Band gap (*E*_g_) of as-deposited and thermally treated Sb_2_S_3_ layers, as estimated assuming direct optical transition and Tauc plots^a^ of optical transmittance spectra of glass/ITO/TiO_2_/Sb_2_S_3_ samples.

	*E*_g_, eV					
Sb/S in sol.	1:3			1:6		
*T*_D_, °C	200	210	220	200	210	220

as-dep.	2.6	2.7	1.8	2.7	1.8	1.8
vac. 170 °C	1.8	1.8	1.8	1.8	1.8	1.8
vac. 200 °C	no layer^b^	1.8	1.8	no layer^b^	1.8	1.8

^a^Supporting Information File 1, Figure S12A,B. ^b^No Sb_2_S_3_ was detected by XRD or Raman.

## Conclusion

The structure, the morphology, and the optical properties of Sb_2_S_3_ layers could be controlled by varying the spray deposition temperature and the molar ratio of precursors in spray solution. Nonuniform, discontinuous layers of polycrystalline Sb_2_S_3_ (*E*_g_ 1.8 eV) were deposited by ultrasonic spray pyrolysis of SbCl_3_/SC(NH_2_)_2_ 1:3 solution at *T*_D_ ≥ 220 °C or 1:6 solution at *T*_D_ ≥ 210 °C on glass/ITO/TiO_2_ substrates in air. Increasing the concentration of the sulfur precursor in spray solution from Sb/S 1:3 to 1:6 reduced the crystallization temperature of Sb_2_S_3_ layers by ≈10 °C. Uniform layers of amorphous Sb_2_S_3_ (*E*_g_ ≈ 2.7 eV, S/Sb 1:3) were deposited on glass/ITO/TiO_2_ substrates in air by ultrasonic spray pyrolysis of Sb/S 1:3 solution at *T*_D_ = 200–210 °C. High quality, uniform, pinhole-free coatings of polycrystalline orthorhombic Sb_2_S_3_ (*E*_g_ 1.8 eV, S/Sb 1.3) with lateral grain size as large as 10 μm were produced by crystallization of amorphous Sb_2_S_3_ layers in vacuum at 170 °C for 5 minutes. Such Sb_2_S_3_ optical coatings are very attractive for future application as low-cost absorber layers in solar cells.

## Experimental

### Materials

Commercial 1.1 mm thick soda-lime glass coated with 150 nm 25 Ω∙sq^−1^ tin doped indium oxide (ITO) from ZSW was used as a substrate. The substrates were rinsed with deionized water, methanol (99.9 vol %), deionized water, dipped in aqueous room temperature H_2_SO_4_ (1 vol %), rinsed again with deionized water, and dried at 105 °C in air.

TiO_2_ was prepared by methods used in our previous papers [7,12]. The TiO_2_ film thickness was ≈80 nm based on SEM images. The Sb_2_S_3_ layers were deposited from 30 mM SbCl_3_ (99 wt %) and SC(NH_2_)_2_ (99 wt %) methanolic (99.9 vol %) solutions at molar ratios of Sb/S 1:3 and Sb/S 1:6. All chemicals were purchased from Sigma-Aldrich and used without any additional processing. The precursor solutions were prepared inside a glovebox with controlled humidity (<14 ppm).

The solutions were ultrasonically nebulized and guided by compressed air at a flow rate of 5 L·min^−1^ onto glass/ITO/TiO_2_ substrates at deposition temperatures of 200, 210, and 220 °C for 20 min (Sb/S 1:3) or 40 min (Sb/S 1:6). After deposition, some of the samples were thermally treated in dynamic vacuum (<4 × 10^−6^ Torr) at 170, 200 or 250 °C for 5 min. The average heating and cooling rate was ≈8 °C·min^−1^.

### Characterization

The elemental composition of the films was determined by energy dispersive X-ray spectroscopy (EDX) using a Bruker spectrometer with ESPRIT 1.8 system at the Zeiss HR FESEM Ultra 55 scanning electron microscope (SEM) operating at an accelerating voltage of 7 kV. The surface and cross-sectional morphologies of the layers were recorded by the same SEM system at an electron beam accelerating voltage of 4 kV.

Unpolarized micro-Raman measurements were conducted at room temperature using a Horiba Jobin Yvon Labram HR 800 spectrometer in backscattering geometry. The laser intensity was attenuated to ca. 143 µW·µm^−2^ over a focal area of Ø 5 µm to prevent oxidation of the Sb_2_S_3_ layers, a common oversight according to Kharbish et al. [21]. Deconvoluted band centers in Raman shift, band intensities and full widths at half maximum (FWHM) were fitted using a Lorentzian function [61].

X-ray diffraction (XRD) patterns were recorded on a Rigaku Ultima IV powder diffractometer in θ-2θ mode (Cu Kα_1_ λ = 1.5406 Å, 40 kV, 40 mA, step 0.02°, 5°/min, silicon strip detector D/teX Ultra). The crystal structure and phase composition were analyzed using Rigaku PDXL 2 software.

Optical total transmittance and total reflectance spectra of glass/ITO/TiO_2_ reference and glass/ITO/TiO_2_/Sb_2_S_3_ samples were measured in the 250–1600 nm range vs air as a reference using a Jasco V-670 UV-VIS-NIR spectrophotometer equipped with a 40 mm integrating sphere and Spectra Manager II software.

## Supporting Information

File 1 Additional XRD, EDX data, SEM images, Lorentzian fitting of Sb_2_S_3_ Raman vibrational bands, and Tauc plots.
