# Supplementary material for: Uniform Sb2S3 optical coatings by chemical spray method
Source: Beilstein J Nanotechnol. 2019 Jan 15;10:198–210. doi: 10.3762/bjnano.10.18 (PMC6350889; doi:10.3762/bjnano.10.18)
Supplement: File 1 — Additional XRD, EDX data, SEM images, Lorentzian fitting of Sb2S3 Raman vibrational bands, and Tauc plots. [file Beilstein_J_Nanotechnol-10-198-s001.pdf]

BEILSTEIN JOURNAL OF NANOTECHNOLOGY

Supporting Information

for

Uniform Sb_2S_3 optical coatings by chemical spray method

Jako S. Eensalu, Atanas Katerski, Erki Kärber, Ilona Oja Acik, Arvo Mere and Malle Krunks

Beilstein J. Nanotechnol. **2019,** *10,* 198–210. doi:10.3762/bjnano.10.18

Additional XRD, EDX data, SEM images, Lorentzian fitting of Sb_2S_3 Raman vibrational bands, and Tauc plots

License and Terms: This is a supporting information file under the terms of the Creative Commons Attribution License (http://creativecommons.org/licenses/by/4.0). Please note that the reuse, redistribution and reproduction in particular requires that the authors and source are credited.
The license is subject to the *Beilstein Journal of Nanotechnology* terms and conditions: (https://www.beilstein-journals.org/bjnano)

Comment S1

The volatilization of Sb_2S_3 layers during vacuum treatment is supported by Sb_2S_3 vapor pressure data we calculated using the Antoine equation and constants provided by Piacente et al. [1]. The partial pressures were calculated by assuming an ideal gas mixture based on Dalton's law [2]. Accordingly, the sum of the partial pressures of all gases in the vacuum chamber was considered equal to the total system pressure.

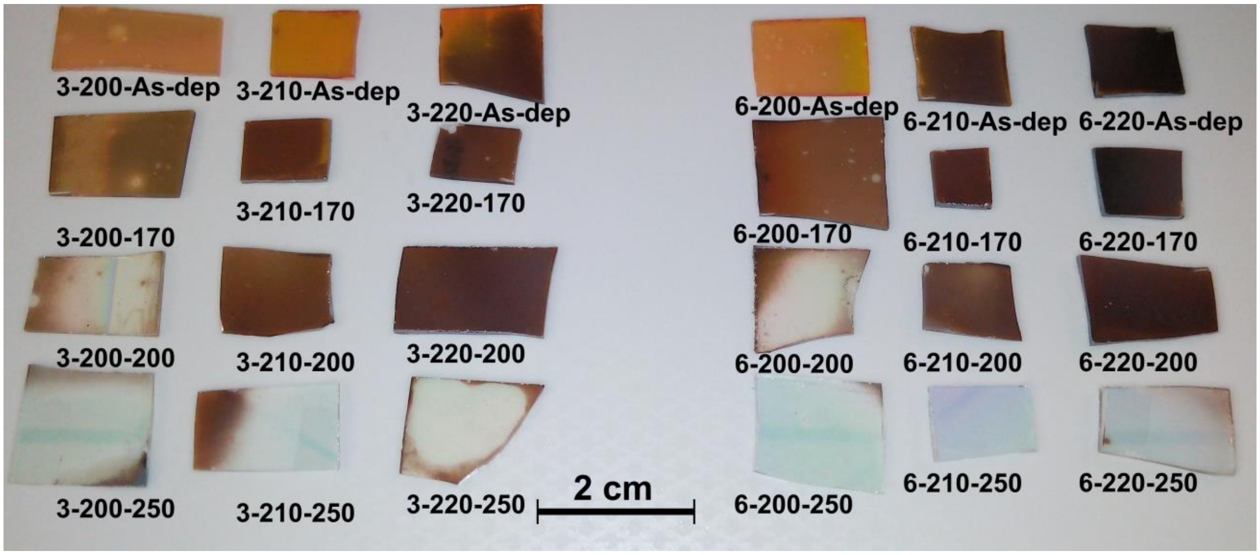

Figure S1: Photograph of as-deposited and vacuum treated glass/ITO/TiO_2/Sb_2S_3 samples on a white background. The appearance of darker Sb_2S_3 layers was lustrous grey at ≈90° viewing angle and brown at smaller viewing angles. As the samples are very reflective, the photograph was taken at an angle to illustrate layer uniformity. Sb_2S_3 layers were deposited at 200 °C, 210 °C, or 220 °C in air from 30 mM Sb/S 1:3 solution for 20 minutes (first, second, and third column on the left), or from 30 mM Sb/S 1:6 solution for 40 minutes (first, second, and third column on the right). Samples were vacuum treated at 170 °C, 200 °C, or 250 °C for 5 min at $<4 \times 10^{-6}$ Torr (second, third, and fourth row, respectively). Sample names in figure: [Sb/S molar ratio in solution]-[Deposition temperature]-[Thermal treatment temperature].

Table S1: Band centers, and uncertainty, as a result of fitting of the Raman spectra of the as-deposited and vacuum heat treated Sb_2S_3 films on glass/ITO/TiO_2 substrate. Sample names in table: [Sb/S molar ratio in solution]-[Deposition temperature]-[Thermal treatment temperature].

Band center, cm^{-1}	281	301	310	R^2 (COD)[a]
13-200-170	281.7±0.1	300.7±0.2	309.0±0.2	0.99942
13-210-As-dep.	281.8±0.1	301.6±0.2	309.6±0.1	0.99891
13-210-170	280.5±0.1	301.2±0.1	309.2±0.1	0.99851
13-210-200	281.2±0.1	302.6±0.2	310.5±0.1	0.99736
13-220-As-dep.	281.5±0.1	301.2±0.2	309.0±0.1	0.99889
13-220-170	280.5±0.1	300.7±0.2	308.7±0.1	0.99844
13-220-200	281.0±0.1	300.1±0.2	308.5±0.2	0.99921
16-200-170	281.6±0.1	301.4±0.1	310.4±0.1	0.99854
16-210-As-dep.	281.9±0.2	298.8±0.5	307.0±0.4	0.99483
16-210-170	281.7±0.1	301.0±0.2	309.7±0.1	0.99882
16-210-200	280.8±0.2	301.2±0.4	309.3±0.2	0.99214
16-220-As-dep.	281.6±0.1	302.0±0.3	310.0±0.1	0.99778
16-220-170	281.0±0.1	300.8±0.2	309.4±0.1	0.99864
16-220-200	280.7±0.1	300.9±0.2	309.1±0.1	0.99778

[a]Coefficient of determination

Table S2: Relative intensities[a], and uncertainty, as a result of fitting of the Raman spectra of the as-deposited and vacuum heat treated Sb_2S_3 films on glass/ITO/TiO_2 substrate. Sample names in table: [Sb/S molar ratio in solution]-[Deposition temperature]-[Thermal treatment temperature].

Band center, cm^{-1}	281	301	310	
	Relative intensity			R^2 (COD)[b]
13-200-170	0.28±0.01	0.54±0.03	0.18±0.02	0.99942
13-210-As-dep.	0.45±0.01	0.37±0.01	0.18±0.01	0.99891
13-210-170	0.67±0.01	0.18±0.01	0.15±0.01	0.99851
13-210-200	0.60±0.01	0.26±0.01	0.15±0.01	0.99736
13-220-As-dep.	0.65±0.01	0.21±0.02	0.14±0.01	0.99889
13-220-170	0.40±0.01	0.42±0.01	0.18±0.01	0.99844
13-220-200	0.57±0.01	0.28±0.02	0.15±0.02	0.99921
16-200-170	0.42±0.01	0.42±0.01	0.16±0.01	0.99854
16-210-As-dep.	0.48±0.03	0.04±0.02	0.48±0.02	0.99483
16-210-170	0.48±0.01	0.35±0.01	0.16±0.01	0.99882
16-210-200	0.58±0.02	0.26±0.03	0.16±0.02	0.99214
16-220-As-dep.	0.69±0.01	0.18±0.02	0.13±0.01	0.99778
16-220-170	0.51±0.01	0.33±0.01	0.16±0.01	0.99864
16-220-200	0.61±0.01	0.23±0.02	0.15±0.01	0.99778

[a]Ratio of peak area to total area of peaks

[b]Coefficient of determination

Table S3: FWHM, and uncertainty, as a result of fitting of the Raman spectra of the as-deposited and vacuum heat treated Sb_2S_3 films on glass/ITO/TiO_2/Sb_2S_3 substrate. Sample names in table: [Sb/S molar ratio in solution]-[Deposition temperature]-[Thermal treatment temperature].

Band center, cm^{-1}	281	301	310	
	FWHM, cm^{-1}			R^2 (COD)[a]
13-200-170	21.5±0.4	21.0±0.5	14.5±0.6	0.99942
13-210-As-dep.	23.2±0.3	15.6±0.3	8.67±0.3	0.99891
13-210-170	22.3±0.3	15.0±0.3	8.04±0.2	0.99851
13-210-200	18.0±0.2	13.0±0.6	6.98±0.3	0.99736
13-220-As-dep.	25.0±0.3	14.7±0.5	9.46±0.3	0.99889
13-220-170	23.6±0.3	14.1±0.6	8.83±0.3	0.99844
13-220-200	22.9±0.4	18.2±0.5	12.5±0.5	0.99921
16-200-170	17.9±0.2	15.9±0.3	8.48±0.3	0.99854
16-210-As-dep.	23.5±1	9.53±3	21.6±0.6	0.99483
16-210-170	21.5±0.3	15.7±0.4	9.7±0.3	0.99882
16-210-200	21.5±0.5	14.5±1	8.57±0.6	0.99214
16-220-As-dep.	23.6±0.3	13.3±0.8	8.51±0.4	0.99778
16-220-170	21.3±0.3	15.5±0.4	9.09±0.3	0.99864
16-220-200	21.9±0.3	13.8±0.6	8.69±0.3	0.99778

Table S4: The positions of detected diffraction peaks and corresponding crystallographic plane indexes of Sb_2S_3 layers deposited on glass/ITO/TiO_2 substrate. Sample names in table: [Sb/S molar ratio in solution]-[Deposition temperature]-[Thermal treatment temperature].

2θ, degree	Plane index, (h k l)
11.1	(1 0 1)
15.8	(2 0 0)/(0 0 2)
17.7	(2 0 1)/(1 0 2)
22.4	(2 0 2)
25.1	(3 0 1)/(1 0 3)
25.8	(1 1 1)
28.2	(2 1 0)/(0 1 2)
28.7	(3 0 2)/(2 0 3)
29.3	(2 1 1)/(1 1 2)
32.5	(2 1 2)
33.5	(3 1 0)/(0 1 3)
34.4	(3 1 1)/(1 1 3)
35.8	(4 0 2)/(2 0 4)

2θ, degree	Plane index, (h k l)
37.2	(3 1 2)/(2 1 3)
39.8	(4 1 0)/(0 1 4)
40.2	(4 0 3)/(3 0 4)
40.7	(4 1 1)/(1 1 4)
43.1	(4 1 2)/(2 1 4)
43.4	(5 0 2)/(2 0 5)
46.9	(4 1 2)/(2 1 4)/(5 1 0)/(0 1 5)
47.2	(5 0 3)/(3 0 5)
47.4	(0 2 0)
47.6	(5 1 1)/(1 1 5)
48.7	(6 0 0)/(0 0 6)
49.4	(6 0 1)/(1 0 6)

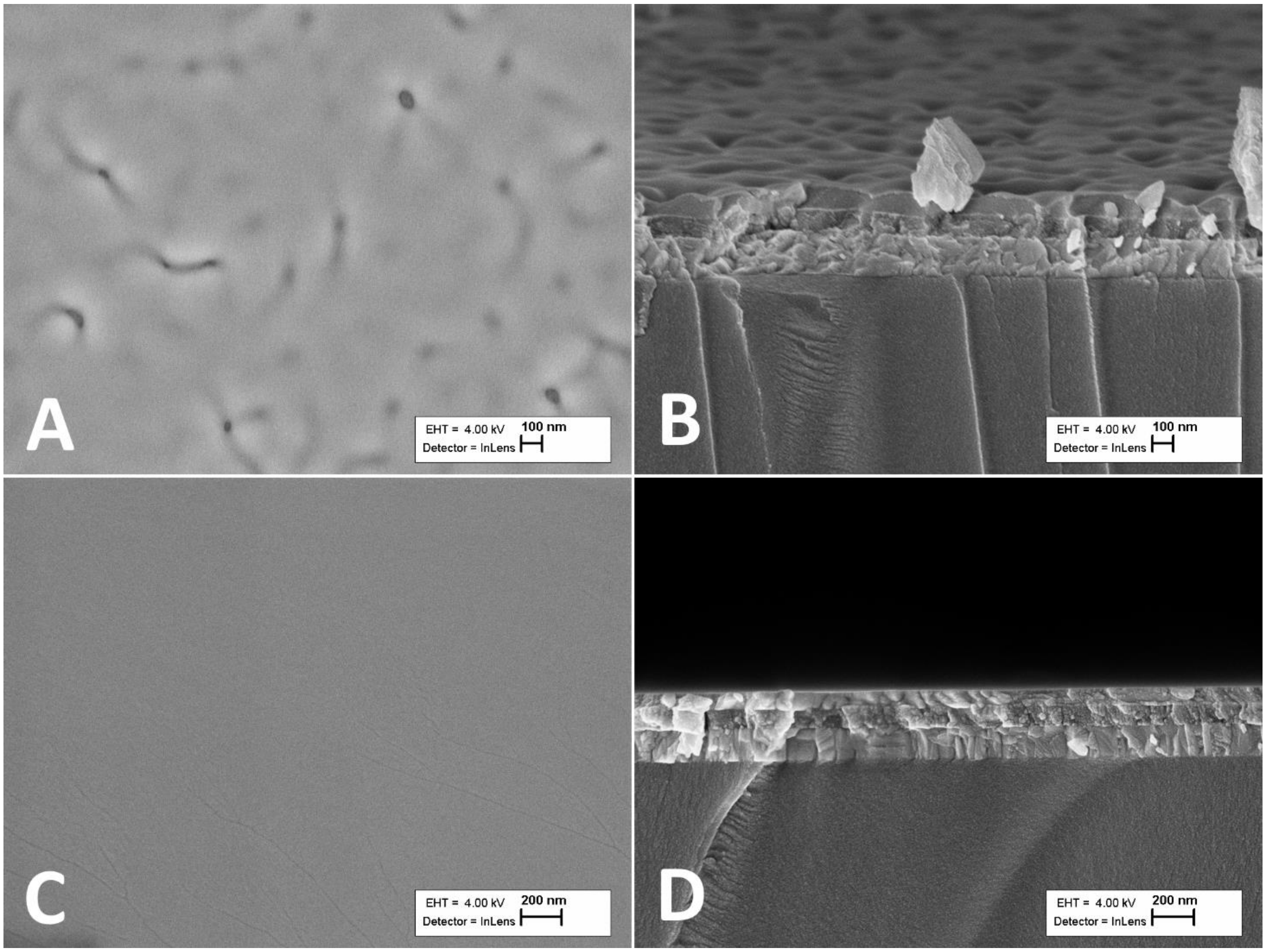

Figure S2: Surface views and cross-sectional views by SEM study of as-deposited (**A, B**) and vacuum heat treated (**C, D**) (170 °C, 5 min, <4 × 10^{-6} Torr) Sb_2S_3 layers. Sb_2S_3 layers were deposited from 30 mM Sb/S 1:3 solution for 20 min at 210 °C in air on glass/ITO/TiO_2 substrate.

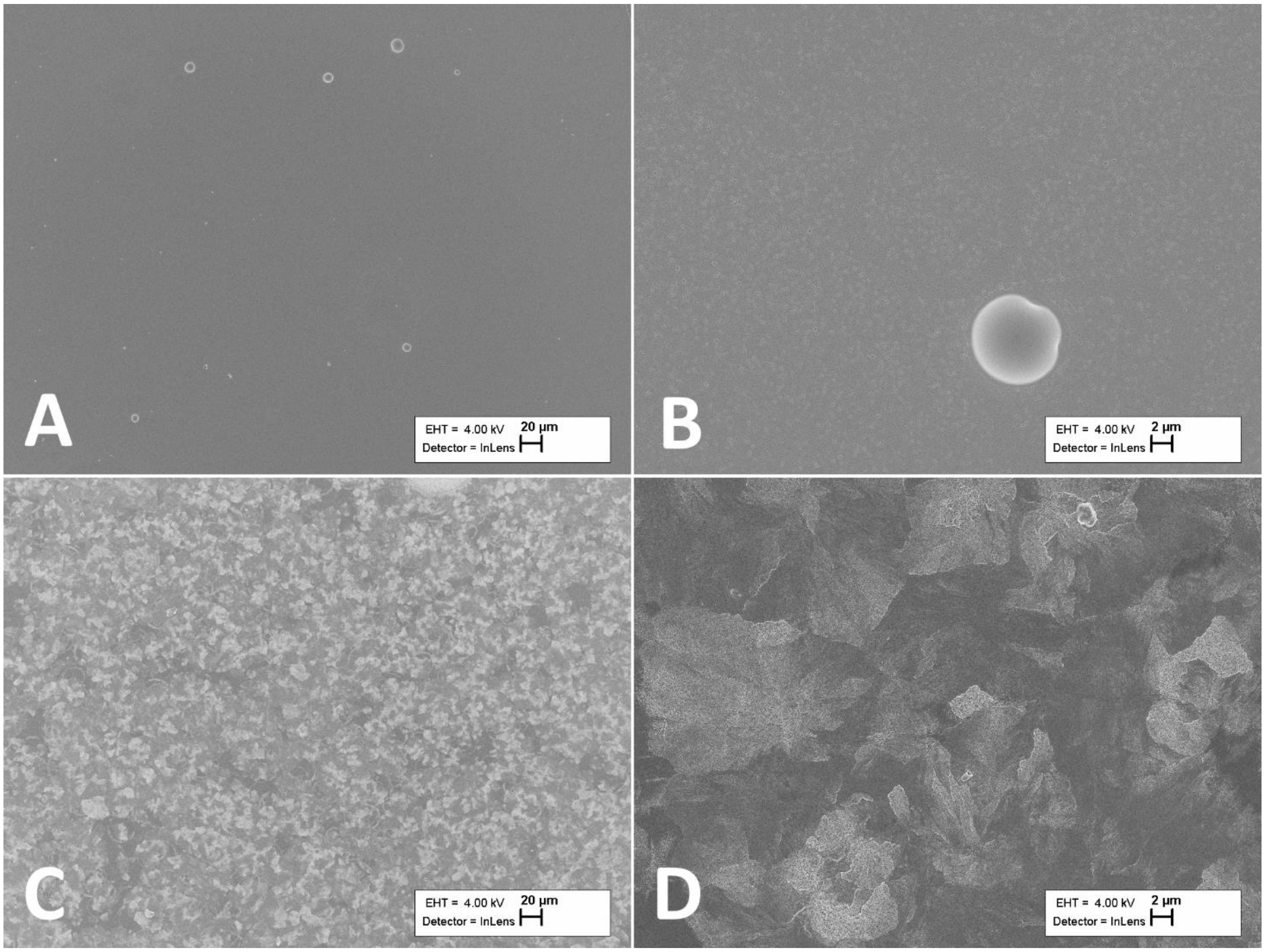

Figure S3: Surface views at different magnifications by SEM study of as-deposited Sb_2S_3 layers. Sb_2S_3 layers were deposited from 30 mM Sb/S 1:3 solution for 20 min at 210 °C (**A, B**) and 220 °C (**C, D**) in air on glass/ITO/TiO_2 substrate.

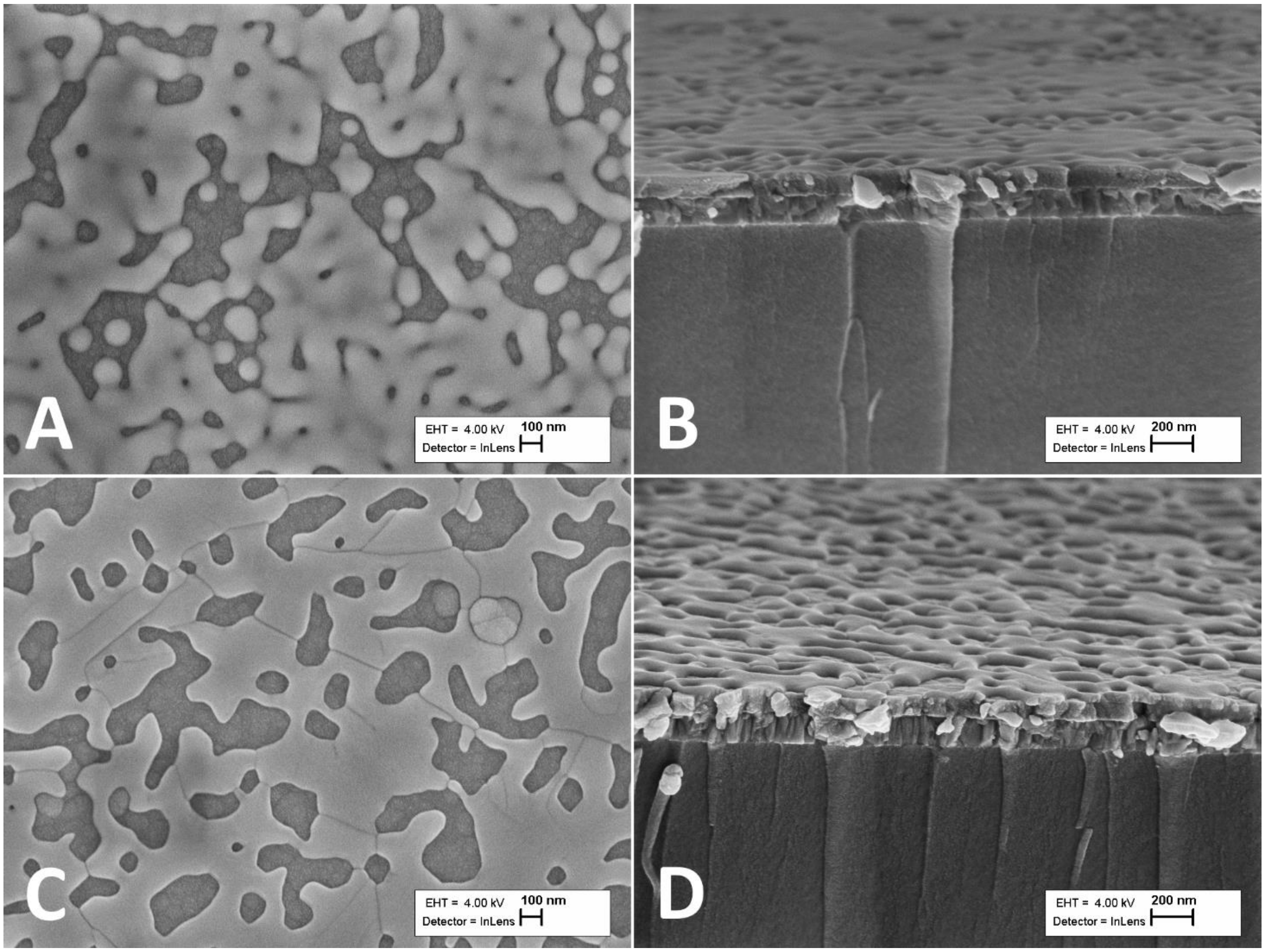

Figure S4: Surface views and cross-sectional views by SEM study of as-deposited (**A, B**) and vacuum heat treated (**C, D**) (170 °C, 5 min, <4 × 10^{-6} Torr) Sb_2S_3 layers. Sb_2S_3 layers were deposited from 30 mM Sb/S 1:6 solution for 20 min at 210 °C in air on glass/ITO/TiO_2 substrate.

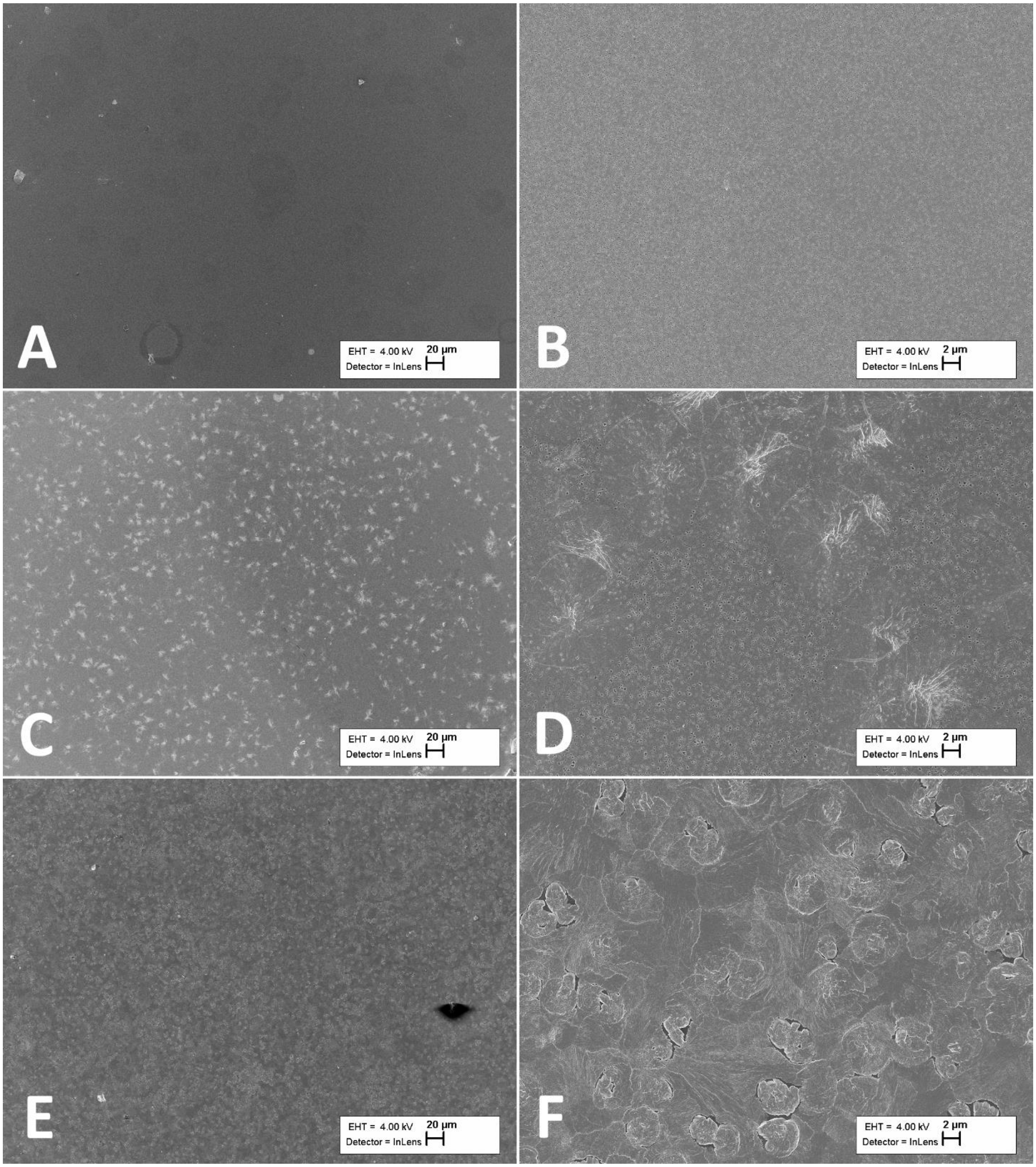

Figure S5: Surface views at different magnifications by SEM study of as-deposited Sb_2S_3 layers. Sb_2S_3 layers were deposited from 30 mM Sb/S 1:6 solution for 40 min at 200°C (**A, B**), 210°C (**C, D**) or 220°C (**E, F**) in air on glass/ITO/TiO_2 substrate.

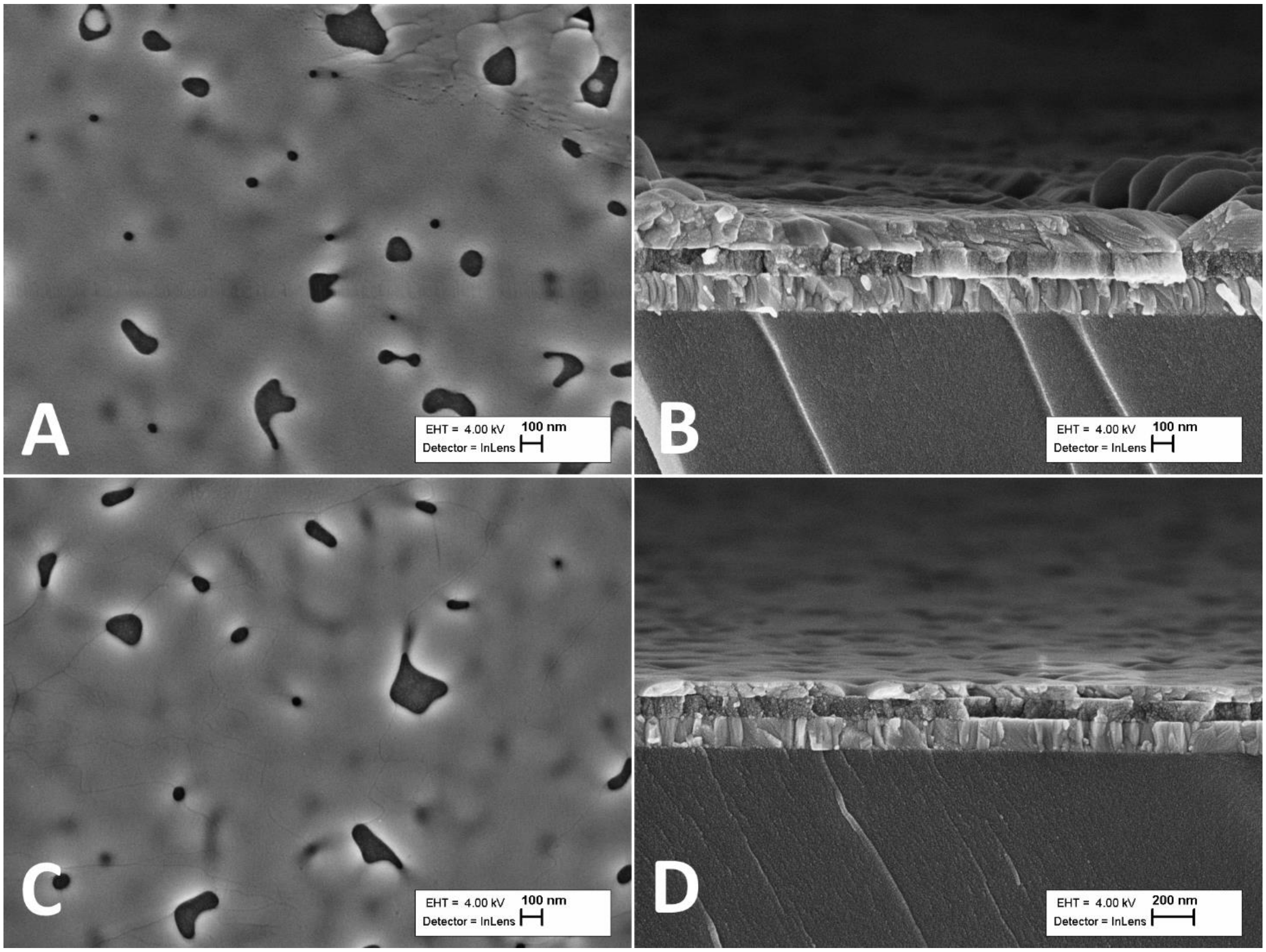

Figure S6: Surface views and cross-sectional views by SEM study of as-deposited (**A, B**) and vacuum heat treated (**C, D**) (170 °C, 5 min, $<4 \times 10^{-6}$ Torr) Sb_2S_3 layers. Sb_2S_3 layers were deposited from 30 mM Sb/S 1:6 solution for 40 min at 210 °C in air on glass/ITO/TiO_2 substrate.

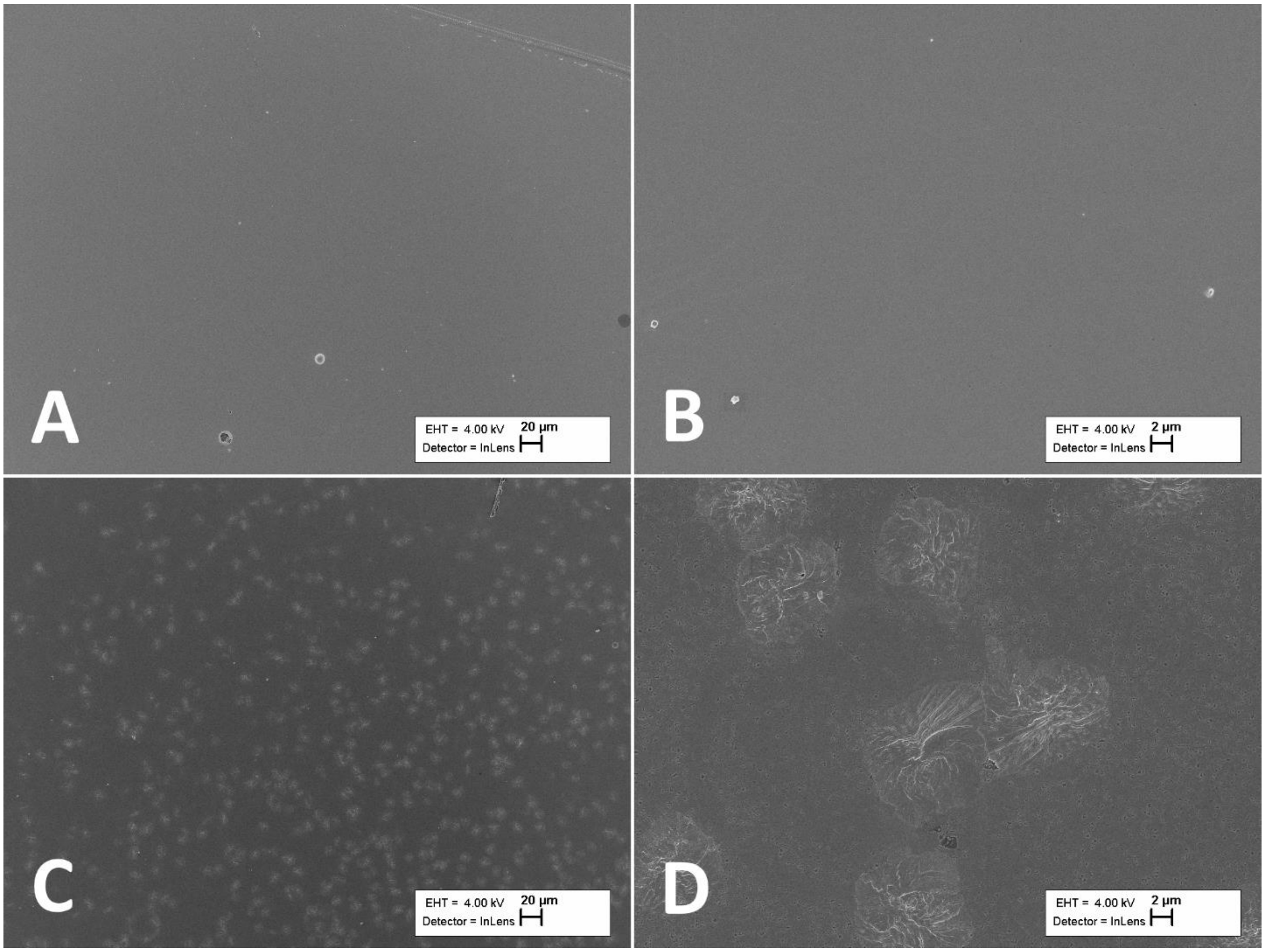

Figure S7: Surface views at different magnifications by SEM study of vacuum heat treated (170 °C, 5 min, <4 × 10^{-6} Torr) Sb_2S_3 layers. Sb_2S_3 layers were deposited from 30 mM Sb/S 1:3 solution for 20 min at 210 °C (**A, B**) or 220 °C (**C, D**) in air on glass/ITO/TiO_2 substrate.

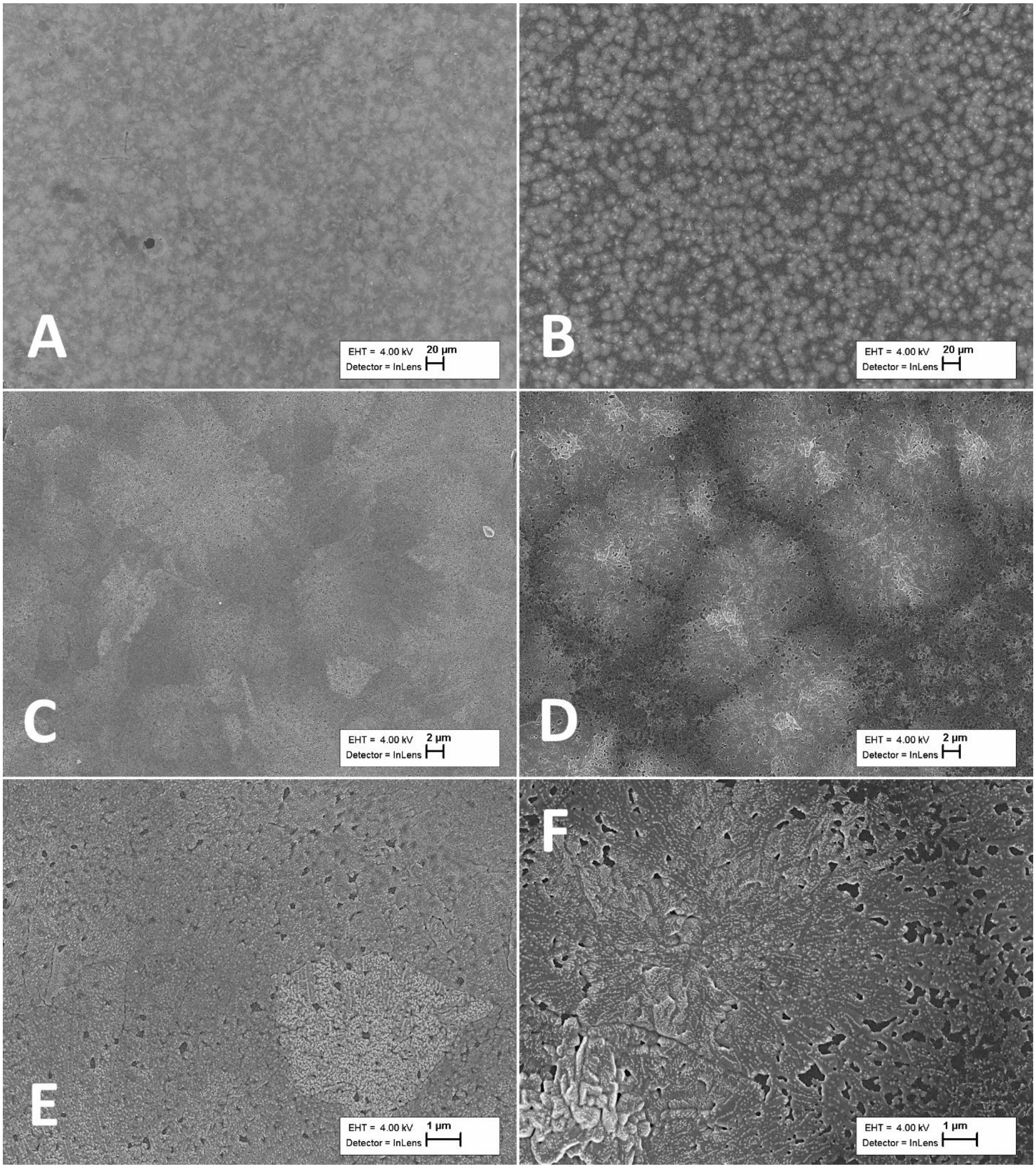

Figure S8: Surface views at different magnifications by SEM study of vacuum heat treated (200 °C, 5 min, <4 × 10^{-6} Torr) Sb_2S_3 layers. Sb_2S_3 layers were deposited from 30 mM Sb/S 1:3 solution for 20 min (**A, C, E**) and from Sb/S 1:6 solution for 40 min (**B, D, F**) at 210 °C in air on glass/ITO/TiO_2 substrate.

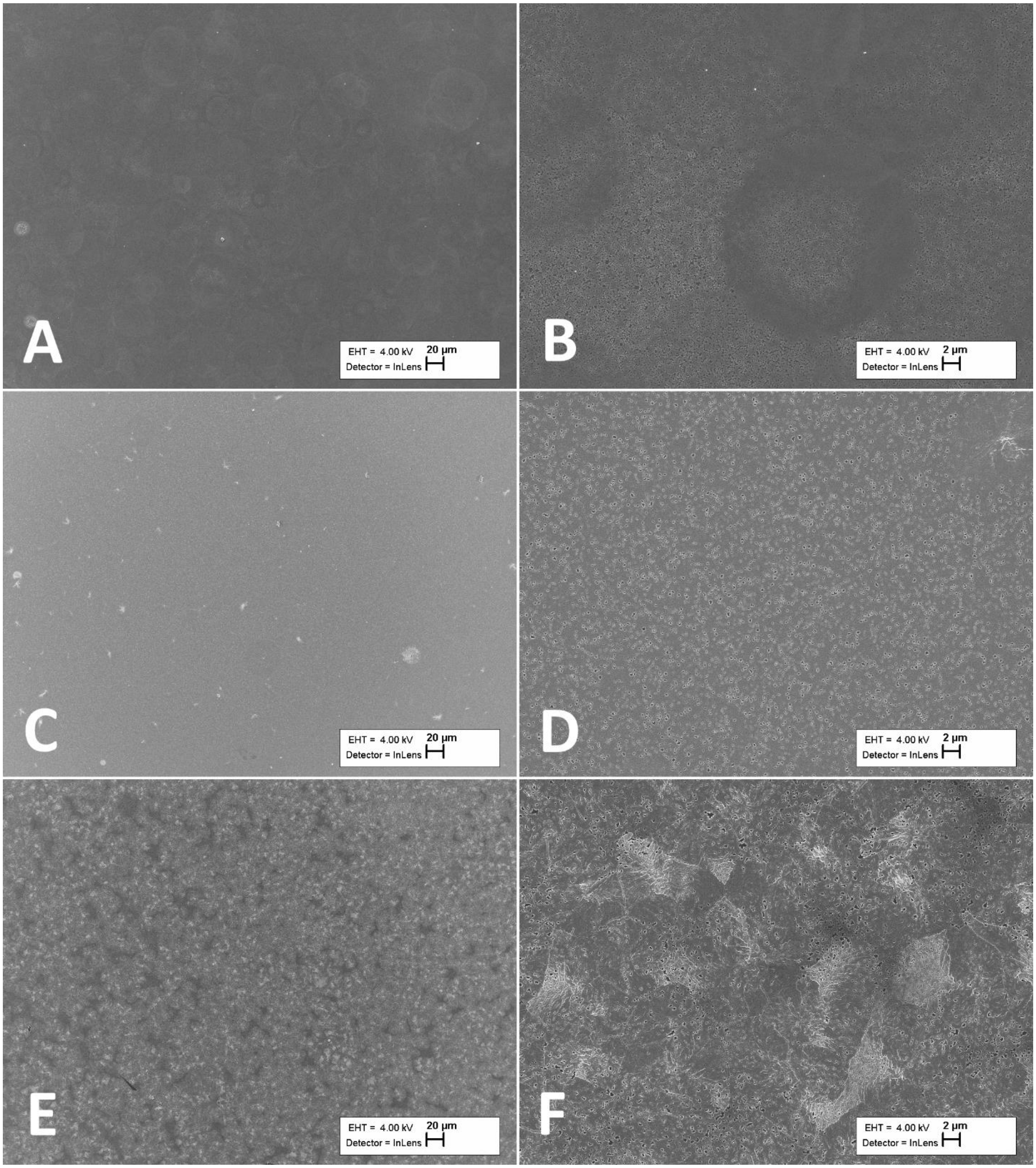

Figure S9: Surface views at different magnifications by SEM study of vacuum heat treated (170 °C, 5 min, <4 × 10^{-6} Torr) Sb_2S_3 layers. Sb_2S_3 layers were deposited from 30 mM Sb/S 1:6 solution for 40 min at 200 °C (**A, B**), 210 °C (**C, D**) or 220 °C (**E, F**) in air on glass/ITO/TiO_2 substrate.

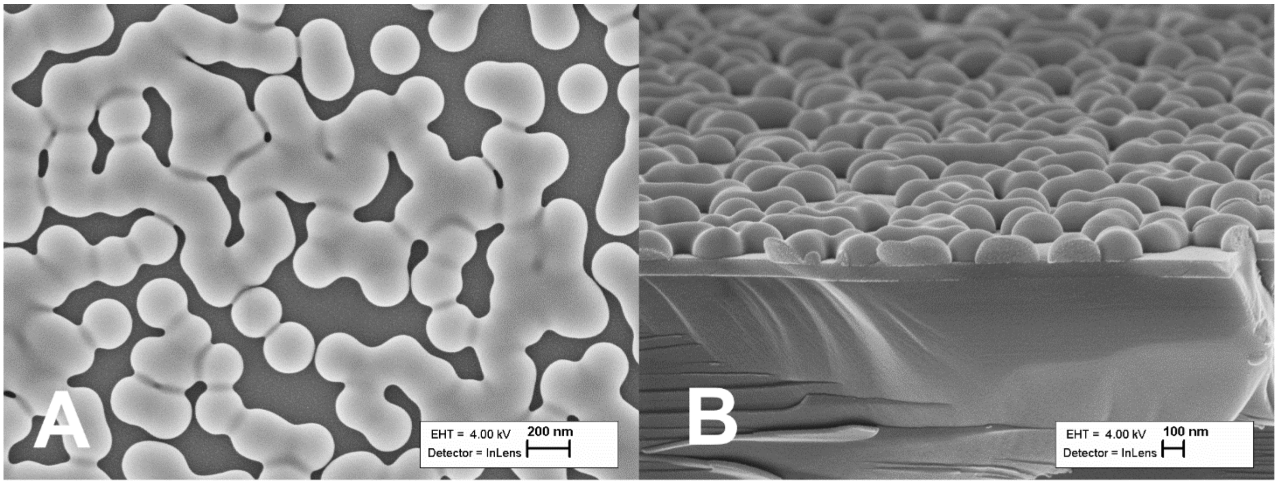

Figure S10: Surface view (**A**) and cross-sectional view (**B**) by SEM study of as-deposited Sb_2S_3 layers. Sb_2S_3 layers were deposited from 30 mM Sb/S 1:3 solution for 20 min at 210 °C in air on Si/SiO_2 substrate.

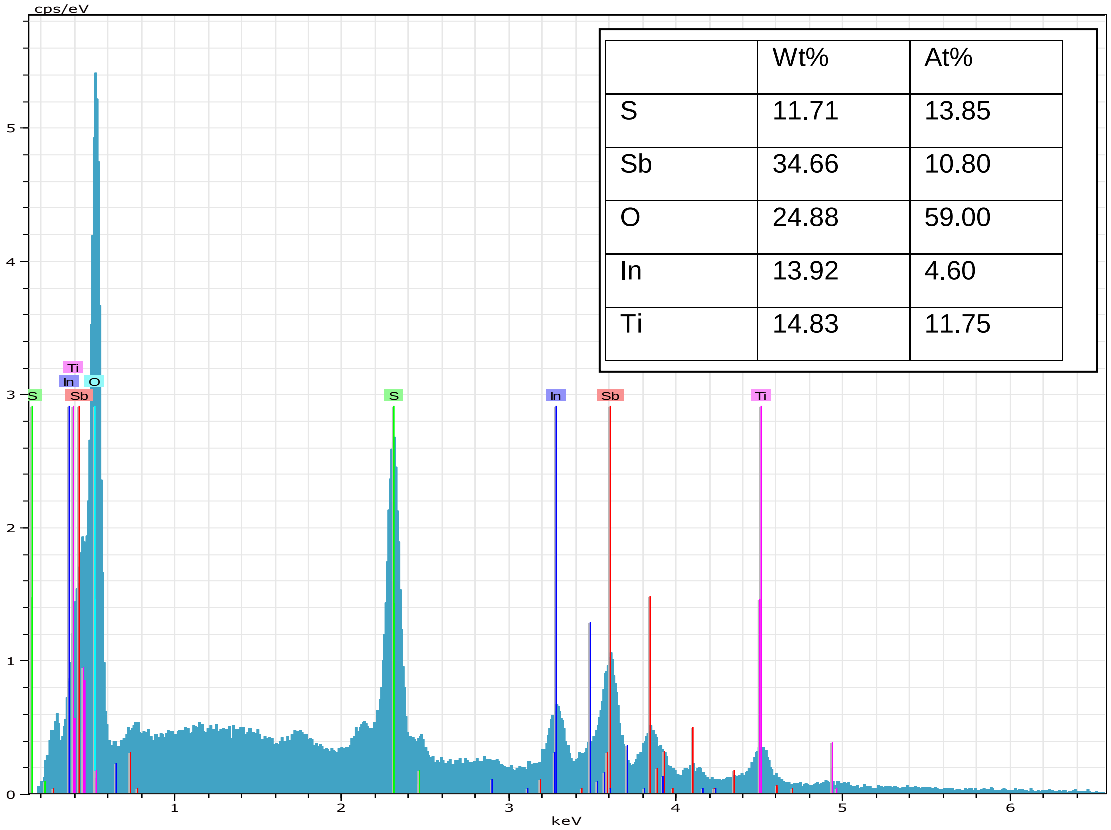

	Wt%	At%
S	11.71	13.85
Sb	34.66	10.80
O	24.88	59.00
In	13.92	4.60
Ti	14.83	11.75

Figure S11: EDX spectrum of glass/ITO/TiO_2/Sb_2S_3 sample vacuum heat treated at 170 °C for 5 min (3-210-170). Sb_2S_3 was deposited from Sb/S 1:3 precursor solution at T_D 210 °C for 20 min.

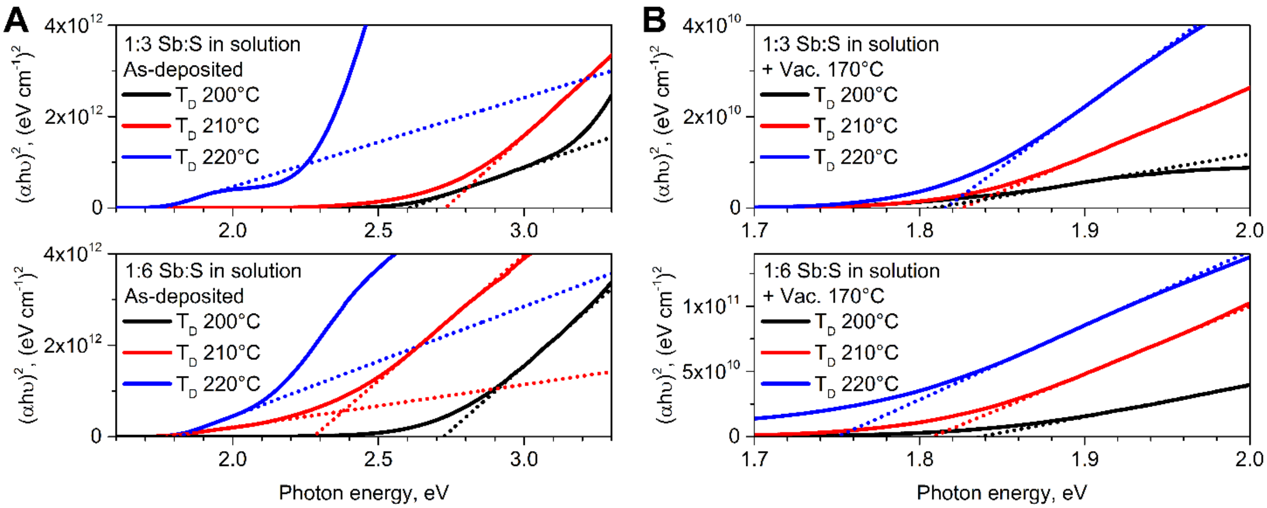

Figure S12: $(\alpha h\nu)^2$ vs $h\nu$ of as-deposited (**A**) and vacuum heat treated (170 °C) (**B**) Sb_2S_3 layers. Sb_2S_3 layers were deposited on glass/ITO/TiO_2 substrate from Sb/S 1:3, or Sb/S 1:6 precursor solution at T_D 200, 210, 220 °C.

References

1. Piacente, V.; Scardala, P.; Ferro, D. *J. Alloys Compd.* **1992**, *178*, No. 1, 101–115.

2. Perry, R. H.; Green, D. W.; Maloney, J. O., Eds.; *Perry's Chemical engineers' handbook*; 7th ed.; McGraw-Hill, 1997.
